# IL-4-Producing Vγ1^+^/Vδ6^+^ γδ T Cells Sustain Germinal Center Reactions in Peyer’s Patches of Mice

**DOI:** 10.3389/fimmu.2021.729607

**Published:** 2021-11-03

**Authors:** Leon Ullrich, Yvonne Lueder, Anna-Lena Juergens, Anneke Wilharm, Joana Barros-Martins, Anja Bubke, Abdi Demera, Koichi Ikuta, Gwendolyn Elena Patzer, Anika Janssen, Inga Sandrock, Immo Prinz, Francesca Rampoldi

**Affiliations:** ^1^ Institute of Immunology, Hannover Medical School, Hannover, Germany; ^2^ Institute for Frontier Life and Medical Sciences, Kyoto University, Kyoto, Japan; ^3^ Institute of Systems Immunology, Hamburg Center for Translational Immunology (HCTI), University Medical Center Hamburg-Eppendorf, Hamburg, Germany

**Keywords:** germinal center, Peyer’s patches, γδ T cells, Vγ1^+^ T cells, IL-4, IgA

## Abstract

The mucosal immune system is the first line of defense against pathogens. Germinal centers (GCs) in the Peyer’s patches (PPs) of the small intestine are constantly generated through stimulation of the microbiota. In this study, we investigated the role of γδ T cells in the GC reactions in PPs. Most γδ T cells in PPs localized in the GCs and expressed a TCR composed of Vγ1 and Vδ6 chains. By using mice with partial and total γδ T cell deficiencies, we found that Vγ1^+^/Vδ6^+^ T cells can produce high amounts of IL-4, which drives the proliferation of GC B cells as well as the switch of GC B cells towards IgA. Therefore, we conclude that γδ T cells play a role in sustaining gut homeostasis and symbiosis *via* supporting the GC reactions in PPs.

## Introduction

Mucosal surfaces of the body are a major entry site for non-self-antigens. The gut-associated lymphoid tissue (GALT) represents the major challenging site for the mucosal immune system, as it has to protect against harmful pathogens, preserve tissue integrity, but should also maintain the tolerance towards commensal microbiota and food antigens (1, 2). Peyer’s patches (PPs) are constantly stimulated by the gut microbiota, which drives the formation of constitutively active germinal centers (GCs) (3, 4). They are formed inside the follicle and are organized into two different anatomical zones, the dark zone (DZ) and the light zone (LZ) (5). B cells in the DZ proliferate extensively (6), maturate in their affinity through somatic hypermutation (SHM), and switch their immunoglobulin (Ig) isotype through class switch recombination (CSR) (7). The gut represents the major induction site for production of IgA (8, 9) in order to maintain the homeostasis of the microbiome (10), and this process is mainly regulated through interleukin-4 (IL-4) (11–13) and transforming growth factor-β (TGF-β) (14, 15). In the LZ, GC B cells are interspersed among a network of follicular dendritic cells (FDCs) (16, 17), which act as an antigen reservoir for the B cells (6). Furthermore, FDCs produce the chemokine CXCL13, sensed by its receptor CXCR5, whose expression attracts GC B cells and T follicular helper (TFH) cells to the LZ (18, 19), where the positive selection of high-affinity GC B cells occurs (20, 21). Positively selected GC B cells can recirculate to the DZ to perform multiple rounds of SHM and selection for high-affinity binding to the antigen (22, 23). These movements between LZ and DZ are regulated by mutual up- and downregulation of CXCR4 and CD86 expression on the surface of the GC B cells (24). Finally, selected high-affinity B cells can leave the GC as plasma cells or memory B cells or re-enter the GC for further diversification (25–27).

γδ T cells are involved in many immunological processes including humoral immunity; however, their role herein is not completely understood (28, 29). In mice, subpopulations of γδ T cells are divided according to their expressed segments in the variable region of the γ-chain (30). Vγ1^+^ T cells are enriched in tissues like spleen and liver (31, 32) and can produce interferon-γ (IFN-γ), IL-4, and IL-13 to help in defense against tumor cells, intracellular pathogens, or extracellular parasites (33–36). In particular, a specific subset of Vγ1^+^ T cells that co-expresses the Vδ6 chain can produce large amounts of IL-4 in the spleen and liver (37). In contrast, Vγ4^+^ and Vγ6^+^ T cells home to different lymphoid and non-lymphoid tissues, where they help in defense against various bacteria through production of IL-17 (38–40).

It has been shown that γδ T cells are able to help B cells for the production of antibodies (28, 29, 41). However, the influence of γδ T cells on the GC reactions in PPs and IgA production is unclear. Here, we focus on the role of γδ T cells in supporting the GC reactions in different mutant mice, which are completely or partially deficient for γδ T cells. We show that γδ T cells are located in the GCs of PPs and that their absence alters not only the development of IgA^+^ GC B cells but also the structure of the GCs. Specifically, we found that a restricted subset of Vγ1^+^ T cells in PPs expressing the Vδ6 chain produced IL-4, thus influencing B cell isotype switch towards IgA.

## Material and Methods

### Animals

*Tcrd*-GDL mice (42), *Tcrd*-H2BeGFP mice (43), and *Tcrd* ^−/−^ mice (44) were bred and housed under specific pathogen-free conditions in the central animal facility at the Hannover Medical School. B6.TCR-Vγ1^−/−^ mice (45) and B6.TCR-Vγ4^−/−^/Vγ6^−/−^ (46) mice were kindly provided by Dr. Rebecca L. O’Brien (National Jewish Health, Denver, USA). Mice were used for experiments at 8 to 12 weeks after birth. All experiments were conducted according to local and institutional guidelines. The study was approved by the Lower Saxony State Office for Consumer Protection and Food Safety, file references: 33.12-42502-04-15/1889, 33.12-42502-04-15/2060, 33.12-42502-04-16/2167, and 33.12-42502-04-19/3289.

### Depletion of γδ T Cells

For conditional depletion of γδ T cells, *Tcrd*-GDL mice were treated i.p. two times, separated by 48 h, with 15 ng of diphtheria toxin (DTx; Merck) per gram body weight (42).

### *Salmonella* Infection

*Tcrd*-GDL mice were injected with DTx for depletion of γδ T cells as described above and compared to non-depleted *Tcrd*-GDL mice. Three days after the first injection, all mice were orally infected with 5×10^9^ *Salmonella typhimurium* (attenuated SL1344 ΔaroA strain, kindly provided by Dr. Dirk Bumann, University of Basel, Switzerland). To reduce confounding effects, co-housed littermates were used. Due to regulatory limitations, these experiments were performed in an S2 restriction area. At day 10, mice were sacrificed, and spleen, mesenteric lymph nodes (mLNs), small intestine (SI), and PPs were collected, homogenized with an Ultra-Turrax (IKA), and plated at different dilutions on LB-media (Lennox, Carl Roth) plates with streptomycin (90 ng/ml; Sigma-Aldrich) overnight at 37°C. The next day, the colony-forming units (CFUs) were counted.

### Anti-γδ TCR Injection

*Tcrd-*H2BeGFP mice were injected i.p. once a week for five times with anti-γδ TCR (clone GL3, 300 µg/mouse) antibodies. One week after the last injection, mice were sacrificed, and PPs were analyzed by flow cytometry.

### Flow Cytometry and Cell Sorting

Mice were sacrificed, and PPs were isolated from the small intestine. Single-cell suspensions from PPs were obtained with the gentleMACS™ Dissociator (Miltenyi Biotec) and filtered through 100 µm cell strainers (Sysmex). Fc-receptors were blocked with 5% anti-FcR antibodies (clone 2.4 G2) in FACS buffer (3% FCS, 40 mM EDTA, PBS) for 30 min on ice. Live/dead cell discrimination was performed by the use of Zombie Aqua Fixable Viability Kit (BioLegend) according to the manufacturer’s instructions. Cell suspensions were stained for flow cytometry by using the following antibodies: antibodies against IgD (clone 11-26c.2a, BV605), CD138 (clone 281-2, BV711), Vγ1.1 (clone 2-11, BV711), CXCR4 [clone 2B11, phycoerythrin (PE)], Vδ6.3/2 (clone 8F4H7B7, PE), CD95 (clone Jo2, PE-Cy7), and CXCR5 (clone 2G8, unlabeled), which were purchased from BD Bioscience; antibodies against CD3 (clone 17A2, Pacific blue), CD4 (clone GK1.5, BV605), CD4 (clone RM4-5, BV650), CD44 (clone IM7, BV605), CD19 (clone 6D5, APC-Cy7), PD-1 (clone 29F.1A12, PE-Cy7), Vγ4 (clone UC3-10A6, APC), and APC-conjugated streptavidin, which were purchased from BioLegend; antibodies against B220 (clone RA3-6B2, eFluor450), GL7 (clone GL-7, eFluor450), NK1.1 (clone PK136, PE-Cy7), CD86 (clone GL1, APC), and PE-conjugated streptavidin, which were purchased from eBioscience; and antibodies against β TCR (clone REA310, APC Vio770), which were purchased from Miltenyi Biotec. The following antibodies against γδ TCR (clone GL3, Alexa Fluor 488 or unlabeled) and against Vγ6 (clone 17D1, unlabeled) were produced in-house with rat hybridoma cell lines. Anti-Vγ7 antibody (clone F2.67, DyLight 650) was provided by P. Pereira (Pasteur Institute, Paris, France). Vγ6 staining was performed as previously described (47). Briefly, after pre-incubation with anti-γδ TCR (clone GL3) for 15 min on ice, anti-Vγ6 (clone 17D1) was added and cells were incubated for further 30 min on ice. Anti-Vγ6 antibodies were detected with anti-IgM (clone RM-7B4, PE; BD Bioscience) antibodies. Samples were acquired using LSRII (BD Bioscience), and data were analyzed using FlowJo software (Version: 10.1, Tree Star). Fluorescence minus one (FMO) controls of Vγ7 TCR, Vδ6.3/2, CD86, CXCR4, and NK1.1 stainings are shown in **Supplementary Figures 1B–E**.

The cell sorting was performed in the Cell Sorting Core Facility of the Hannover Medical School by using the FACSAria Fusion (BD) or the FACSAria II (BD). γδ T cells of *Tcrd*-H2BeGFP mice were sorted after staining with fluorophore-conjugated anti-β TCR and GFP expression; while B cells after staining with anti-B220 antibodies. The purity of sorted populations was 95–99%.

### BrdU Incorporation

Three-week γδ T cell–depleted and non-depleted *Tcrd*-GDL mice were injected i.p. with bromodeoxyuridine (BrdU; 1 mg/ml). 2 h after the injection, PPs of mice were collected, and cells were stained with a BrdU Flow Kit (Cat. 552598, BD Bioscience) according to the manufacturer’s instructions. Briefly, cells were firstly stained with surface markers, fixed, and permeabilized. After treatment with DNase I, cells were stained with anti-BrdU antibodies conjugated with APC for 30 min at room temperature and then analyzed by flow cytometry.

### IL-4 Secretion Assay

Ninety-six-well plates (Nunc) were coated overnight with monoclonal antibodies (mAb) anti-CD3 (1 µg/ml, clone 17A2, unlabeled, in-house produced by rat hybridoma cell lines) and CD28 (1 µg/ml, clone 37.51, unlabeled, purchased from eBioscience) to stimulate γδ T cells. Single-cell suspensions of PPs were obtained like previously described. Then 4×10^6^ cells/ml were resuspended in RMPI 1640 (10% FCS, 1% Pen/Strep, 1% L-Glutamine) and incubated at 37°C for 2 h. After stimulation, antibodies for surface staining were added for 30 min on ice together with anti-FcR antibodies (clone 2.4 G2) and live/dead cell discrimination agent (Zombie Aqua Fixable Viability Kit, BioLegend). Next, IL-4 secretion assay (Miltenyi Biotec) was performed according to the manufacturer’s instructions. Briefly, cells were incubated for 5 min with IL-4 catch antibodies on ice and afterwards for further 45 min at 37°C to secrete IL-4. The secreted IL-4 was then detected with PE-labeled IL-4 detection antibodies for 10 min on ice. After washing, cells were acquired and data analysis was performed using Flowjo software (Version: 10.1, Three Star). Gating strategy and FMO control of IL-4 are shown in **Supplementary Figure 1A**.

### *In Vitro* Culture of PP B Cells

After cell sorting, γδ T and B cells isolated from PPs were resuspended in cell culture media (IMDM, 10% FCS, 1% penicillin-streptomycin, 1% L-glutamine, and 50 µM ß-mercaptoethanol) in a ratio of 1:10 (γδ T cells: B cells) and transferred into a ninety-six-well U bottom plate (Sarstedt). The total number of cells was 55,000 per well in a volume of 100 µl of cell culture media. For the induction of IgA isotype switch (48), the following cytokines were added or not to the media: murine IL-4 (100 ng/ml; PeproTech), murine IL-5 (1 ng/ml; PeproTech), human TGF-β (1 ng/ml; PeproTech), and *Escherichia Coli* LPS (10 µg/ml, Sigma-Aldrich). Cells were incubated for three days at 37°C and 5% CO_2_. Afterwards, murine IL-6 (1 ng/ml; PeproTech) was added to the culture media, and cells were incubated for additional 3 days before being analyzed.

### Hematoxylin and Eosin Staining

Five centimeters of the proximal part of the small intestine of the mice were rolled and fixed in 2% buffered formalin for 4 h and embedded in paraffin. Sections (5 µm) were stained with hematoxylin and eosin (H/E; Sigma-Aldrich). After washing and mounting, slides were acquired with a Zeiss Axioscan.Z1 with 10× objective, and images were analyzed by Zen Blue software (Version: 2.3, Zeiss).

### Immunohistology

A cut of the proximal, medial, and distal parts of the PPs were taken, and frozen sections (8 µm) were fixed in ice-cold acetone for 10 min. After rehydration, sections were incubated with 10% rat sera or 5% mouse sera and anti-FcR antibodies (clone 2.4 G2) in TBS-T for 15 min at RT according to the staining. For GC staining, sections were incubated for 1 h at RT with the following antibodies: anti-Ki-67 (1:100, clone SolA15, FITC, eBioscience), anti-GL7 (1:100, clone GL7, Alexa Fluor 647, BioLegend), anti-CD86 (1:100, clone GL1, APC, eBioscience), and anti-CXCR4 (1:100, clone 2B11, PE, BD Bioscience). For the FDC staining, sections were incubated for 1 h at RT with anti-FDC-M1 (1:100, clone FDC-M1, unlabeled, BD Bioscience) or anti-CD35 (1:100, clone 8C12, BV421, BD) antibodies. Together with the anti-CD35 antibodies, the following antibodies were used: anti-GL7 (1:100, clone GL7, Alexa Fluor 647, BioLegend) and anti-IgD (1:100, clone HB250, Cy5, home-made); for nuclei visualization, propidium iodide was used (4 min; 1 µg/ml, Sigma-Aldrich). To visualize the FDC-M1 antibodies, sections were stained for 1 h at RT with mouse anti-rat IgG (H+L) F(ab’)_2_ fragment (1:200, Cy3, Jackson ImmunoResearch). After blocking with 10% rat sera for 15 min at RT, sections were then stained for 1 h with anti-IgD (1:100, clone HB250, Cy5) in-house produced with rat hybridoma cell lines. All sections, except the ones stained with anti-CD35 antibodies, were stained with DAPI 1µg/ml (Sigma-Aldrich) for 3 min. Afterwards, they were mounted with FluorSave reagent (Merck) and treated similarly for high comparability. For analysis of the marker expression, composite pictures of whole PPs were acquired using Zeiss Axioscan.Z1 with a 10× objective. For analysis of Ki-67, CXCR4, CD35, CD86, GL7, and FDC-M1 expression, the same adjustment was applied to all pictures using Zen Blue software (Zeiss), and GCs were selected and extracted based on their DAPI signal for further analysis with ImageJ (Version: 1.52p). Areas of Ki-67, CXCR4, CD35, CD86, and FDC-M1 staining were measured automatically using a self-written macro. In short, GC area was selected manually based on DAPI signal. Only this GC region was then used for automatic analysis of expression of Ki-67, CXCR4, CD35, CD86, and FDC-M1. Single channels were binarized, and a fixed threshold was applied before signal area was measured automatically. The calculated areas for the different markers were normalized to GC size.

### Confocal Microscopy

Sixteen µm sections of PPs were cut and fixed in ice-cold acetone for 2 min. After rehydration, sections were blocked with 5% rat sera and anti-FcR antibodies (clone 2.4 G2) in PBS-T for 30 min at 37°C. For the staining, sections were incubated for 30 min at 37°C with the following antibodies: anti-GL7 (1:100, clone GL7, Alexa Fluor 647, BioLegend) and anti-IgD (1:100, clone HB250, Cy5, home-made). All sections were then stained with DAPI 1µg/ml (Sigma-Aldrich) for 3 min and mounted with FluorSave reagent (Merck).

For the acquisition of confocal z-stack images, a Zeiss LSM 980 confocal microscope (Zeiss) with 63× oil objective lens was used at the optimal interval of 1 µm to the z-direction. The areas of GCs and B cell follicles were measured with Zen Blue software (Zeiss). The number of γδ T cells (calculated based on the expression of eGFP) was normalized to the area of GC or B cell follicle. Z-stack images and AVI videos were generated using Imaris software (Version 9.5.1, Bitplane) at 24 frames per second. Gamma values of DAPI were adjusted for better visibility.

### Isolation of Igs From Feces for ELISA

Feces were collected and mixed in protein isolation buffer [1 mM PMSF (Carl Roth), 1× protein inhibitor solution (Roche) in PBS] until a homogenous solution was formed. The tubes were centrifuged at 5,000 *g*, 4°C for 10 mins, and total protein concentration was measured by using Advanced Protein Assay reagent (Cytoskeleton) according to the manufacturer’s instructions. Samples were frozen at −20°C until analysis.

Ninety-six-well plates (Nunc) were coated with goat anti-mouse/human ads-UNLB (1:3,000 in PBS; SouthernBiotech), overnight at 4°C. Unspecific binding sites were blocked with 3.5% BSA (Biomol) in PBS for 1 h at 37°C. Samples were adjusted to a concentration of 8 µg/µl with PBS. As reference for quantification, serial dilutions of Ig standard [mouse IgA-UNLB (1–0.0005 µg/ml); SouthernBiotech] were established. Standard and samples were incubated for 3 h at RT in the dark. Afterwards, HRP-coupled antibodies [goat anti-mouse IgA (1:8,000, SouthernBiotech)] were incubated for 1 h at RT in the dark. The enzymatic reaction was started by adding TMB substrate (Thermo Fisher Scientific) and was stopped with 0.5M H_2_SO_4_ (Carl Roth). Absorbance was measured at 450 nm with reduction at 595 nm using a SpectraMax iD3 (Molecular Devices).

### Intestinal Permeability Assay

Mice were fasted for 3 h, and then fluorescein isothiocyanate (FITC)-coupled dextran (4,000 Da, Sigma-Aldrich) was administered by gavage (600 mg/kg body weight). After 1 h, mice were sacrificed and blood was taken from the retro-orbital sinus of the eye. Plasma was obtained by centrifugation, diluted 1:2 with PBS, and added to a plate with black background (Costar) in duplicates. A standard curve of FITC-dextran (25–0.024 µg/ml) was added as a reference for quantification. Fluorescent signal was measured at 485 nm excitation and 528 nm emission wavelength using a SpectraMax iD3 (Molecular Devices).

### 16S rRNA Gene Sequencing

Feces was used to extract DNA with the QiAamp Fast DNA Stool Kit (Qiagen) according to the manufacturer’s instructions.

Total DNA of samples was processed following the protocol for 16S Metagenomic Sequencing Library Preparation (Illumina, Part # 15044223 Rev. B). In brief, PCR of bacterial 16S rRNA (V3-V4 regions) was performed to create a single amplicon of approximately 460 bp. Illumina sequencing adapters and dual-index barcodes were added to the amplicons using the Nextera XT Index Kit (Illumina). Paired-end sequencing was carried out on Illumina MiSeq platform (Illumina) with MiSeq Reagent Kit V3 (Illumina).

### 16S Metagenomic rRNA Gene Sequencing Data Analysis

Illumina BaseSpace 16S Metagenomics app (Illumina) was used to generate a classification of reads at taxonomic levels from kingdom to species. The classification step uses a proprietary algorithm that provides species-level classifications for paired-end reads, involving matching short subsequences of the reads to a set of 16S reference sequences (Greengenes database).

### Statistical Analysis

Results from experiments were analyzed by GraphPad Prism (Version: 8.4.3, GraphPad Software). The values presented are mean ± SEM of *n* independent experiments. Differences between individual groups were analyzed as indicated in figure legends by either unpaired Students *t*-test or one-way ANOVA followed by Tukey’s multiple comparison post-test. P values < 0.05 were considered to be significantly different.

## Results

### γδ T Cells Localize in GCs of PPs

**Figure 1:**
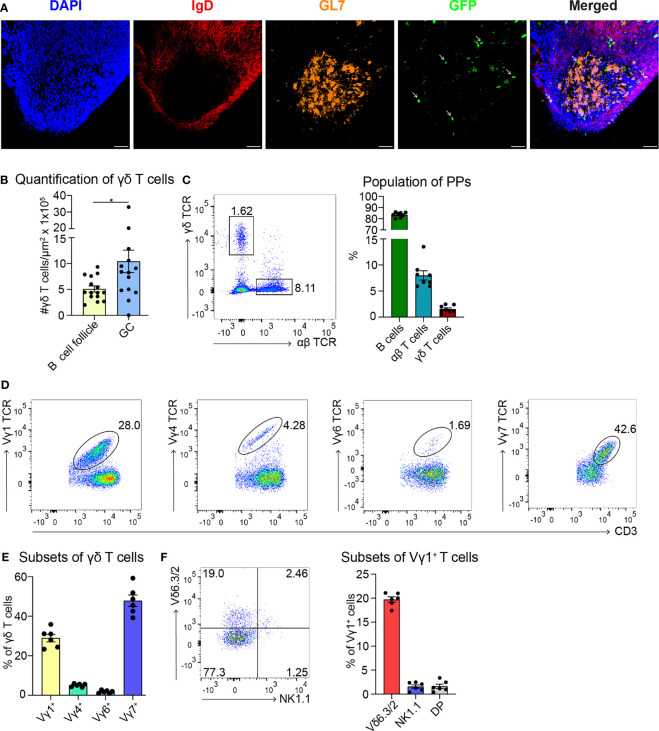
Most of the γδ T cells localize in GCs of PPs and express the Vγ1^+^ TCR. **(A)** Confocal fluorescence microscopy of frozen sections of PPs from *Tcrd*-H2BeGFP mice stained with anti-IgD (red), anti-GL7 (orange), and DAPI (blue) to detect the nuclei. GFP-expressing γδ T cells (green) are highlighted with an arrow. Scale bar, 50 µm. **(B)** γδ T cells inside the B cell follicles (IgD^+^) and germinal centers (GC; IgD^−^, GL7^+^) were quantified. For the quantification, sections of PPs were analyzed from different mice. Each dot represents an individual cut. Bar graph, mean ± SEM. n = 5 mice. **(C)** FACS analysis of B cells (CD19^+^, TCRβ^−^), αβ T cells (CD19^−^, CD3^+^, TCRβ^+^), γδ T cells (CD19^−^, CD3^+^, GFP^+^) in PPs from *Tcrd*-H2BeGFP mice. Bar graph, mean ± SEM. n = 8. **(D)** FACS analysis of Vγ1 TCR, Vγ4 TCR, Vγ6 TCR, and Vγ7 TCR gated on γδ T cells (TCRβ^−^, CD3^+^, GFP^+^) of PPs from *Tcrd*-H2BeGFP mice. **(E)** Quantification of Vγ1 TCR, Vγ4 TCR, Vγ6 TCR, and Vγ7 TCR expressed on γδ T cells (TCRβ^−^, CD3^+^, GFP^+^) of PPs from *Tcrd*-H2BeGFP mice. Bar graph, mean ± SEM. n = 6. **(F)** FACS analysis of Vδ6.3/2, and NK1.1 gated on Vγ1^+^ T cells (TCRβ^−^, CD3^+^, GFP^+^, Vγ1 TCR^+^) of PPs from *Tcrd*-H2BeGFP mice. Bar graph, mean ± SEM. n = 6. Each dot represents an individual mouse. *P < 0.05.

**Figure 2:**
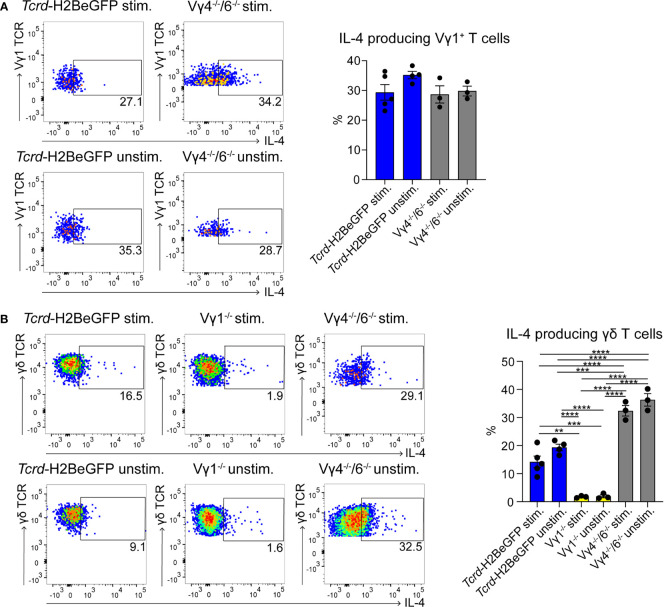
γδ T cells represent a major source of IL-4 in PPs. **(A, B)** IL-4^+^ γδ T cells were measured by flow cytometry with an IL-4 secretion assay. PPs from *Tcrd*-H2BeGFP, Vγ1^−/−^, and Vγ4^−/−^/Vγ6^−/−^ mice were stimulated with mAbs directed against CD3 (1 µg/ml) and CD28 (1 µg/ml) for 2 h and compared to the unstimulated controls. **(A)** FACS analysis of IL-4^+^ Vγ1^+^ T cells in PPs of *Tcrd*-H2BeGFP and Vγ4^−/−^/Vγ6^−/−^ mice gated on Vγ1^+^ T cells (CD19^−^, TCRβ^−^, CD3^+^, GFP^+^, Vγ1 TCR^+^). Bar graph, mean ± SEM. n = 3–5 per group. ANOVA test was applied with Tukey *post-hoc* test. **(B)** FACS analysis of IL-4^+^ γδ T cells of PPs from *Tcrd*-H2BeGFP, Vγ1^−/−^, and Vγ4^−/−^/Vγ6^−/−^ mice gated on γδ T cells (CD19^−^, TCRβ^−^, CD3^+^, GFP^+^). Bar graph, mean ± SEM. n = 3–5 per group. ANOVA test was applied with Tukey *post-hoc* test. Each dot represents an individual mouse. **P < 0.01, ***P < 0.001, ****P< 0.0001.

To date, few data are available regarding the localization of γδ T cells in PPs (49). To investigate this further, we took advantage of a mouse model expressing histone2B-coupled eGFP in the nuclei of all γδ T cells (43). We performed confocal z-stack imaging on individual sections of PPs and quantified γδ T cells in the B cell follicles and in the GCs. We found that significantly more γδ T cells are located in the GCs compared to the B cell follicles (**Figures 1A, B**, and **Supplementary Movie 1**). We next examined the different populations of lymphocytes in PPs, of which 83% were B cells, 8% were αβ T cells, and 1.5% were γδ T cells (**Figure 1C**). Among γδ T cells, we found that 29% were Vγ1^+^ T cells, 5% were Vγ4^+^ T cells, 1.5% were Vγ6^+^ T cells, and to our surprise, 48% were Vγ7^+^ T cells, most probably co-isolated intraepithelial γδ T cells (**Figures 1D, E**). We then focused on Vγ1^+^ T cells and specifically analyzed the expression of the markers NK1.1 and Vδ6.3/2, since they define different functional subsets of Vγ1^+^ T cells. Vγ1^+^/Vδ6^+^ T cells represent a distinct subpopulation in spleen and liver of mice that can produce higher amounts of IL-4 than IFN-γ, whereas, Vγ1^+^/NK1.1^+^ T cells can produce more IFN-γ than IL-4 and share functional properties with invariant NKT cells (37, 50, 51). In PPs, approximately 20% of Vγ1^+^ T cells expressed the Vδ6.3/2 chain, but only 1.5% of them expressed the NK1.1 marker, leading us to hypothesize that a considerable proportion of γδ T cells in PPs are able to produce IL-4 (**Figure 1F**). To investigate the ability of Vγ1^+^ T cells to produce IL-4, we thus measured the secretion of this cytokine *in vitro*. We found that ca. 30% of unstimulated and stimulated Vγ1^+^ T cells isolated from *Tcrd*-H2BeGFP mice and Vγ4^−/−^/Vγ6^−/−^ mice produced high levels of IL-4 (**Figure 2A**). We further corroborated these data by comparing γδ T cells from *Tcrd*-H2BeGFP mice and Vγ4^−/−^/Vγ6^−/−^ mice (possessing Vγ1^+^ T cells) with Vγ1^−/−^ mice (possessing Vγ4^+^ and Vγ6^+^ but not Vγ1^+^ T cells) (**Figure 2B**). In contrast to the other strains, γδ T cells from Vγ1^−/−^ mice were not able to secrete IL-4, indicating that the Vγ1^+^ T cells are the major producer of IL-4 among γδ T cells (**Figure 2B**). Notably, the stimulation with mAbs directed against CD3 and CD28 did not make a difference for γδ T cells, probably because they are continuously activated by the gut microbiota.

TFH cells play a key role during GC reactions by providing help to the B cells and by producing IL-4 (52). Therefore, we investigated these cells, but no differences in TFH cell frequencies in PPs could be observed (**Supplementary Figure 2A**). Moreover, their ability to produce IL-4 was not compromised in any of the mutant mice (**Supplementary Figure 2B**). Interestingly, the number of TFH cells producing IL-4 was lower compared to the number of the IL-4-producing Vγ1^+^ T cells (**Figure 2A** and **Supplementary Figure 2B**), suggesting that these two cells represent together the main source of IL-4 in the PPs.

Taken together, γδ T cells could be detected in high frequencies in GCs of PPs. These were mainly Vγ1^+^ T cells co-expressing the Vδ63/2 chain and able to secrete IL-4.

### Depletion of γδ T Cells Does Not Alter the Permeability and the Microbiota of the Gut

Next, we applied a newly established mouse model for conditional depletion of γδ T cells with DTx, namely, the *Tcrd*-GDL mice (42). After injection of DTx, all γδ T cells are depleted, but after 3 weeks they start to regenerate (**Supplementary Figure 3A**). To exclude the possibility that γδ T cell depletion might compromise the permeability of the gut, we first evaluated possible differences in the structure of the gut cells and epithelium. H/E staining of the small intestine did not show any remarkable architectural differences (**Supplementary Figure 4A**). To control potential changes in gut permeability, we performed a FITC-dextran absorption assay, which did not show significant changes in the permeability in *Tcrd*-GDL mice at 8 days after γδ T cell depletion when compared to control *Tcrd*-H2BeGFP mice (**Supplementary Figure 4B**). These findings implicate that no increased translocation of pathogens would occur across the epithelial intestinal barrier.

We then asked whether γδ T cell depletion might influence the gut microbiota. To investigate this, we performed 16S metagenomic analysis of the bacteria from feces of non-depleted and γδ T cell-depleted (8 days, 3 weeks, and 8 weeks) *Tcrd*-GDL mice. However, species diversity, as estimated by the Shannon index, was not significantly different between the groups (**Supplementary Figure 4C**). Moreover, on a compositional level, the distribution of the families across the samples was very similar (**Supplementary Figure 4D**).

In conclusion, absence of γδ T cells did compromise neither the structure and the function of the small intestinal mucosa nor the composition of the intestinal microbiota of depleted *Tcrd*-GDL mice.

### γδ T Cells Influence the Development of IgA^+^ GC B Cells and the Structure of the GCs

**Figure 3:**
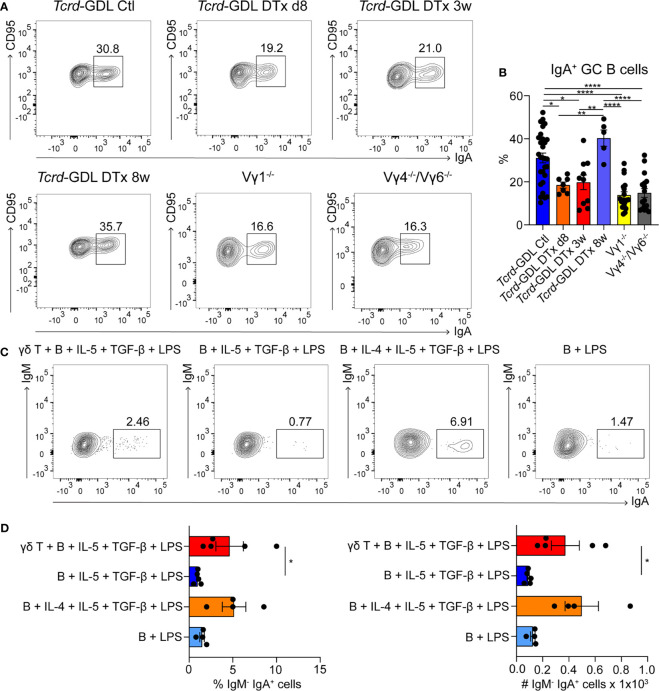
γδ T cells are involved in the generation of IgA^+^ GC B cells. **(A, B)** PPs were isolated from non-depleted and γδ T cell–depleted (8 days, 3 weeks, and 8 weeks) *Tcrd*-GDL, Vγ1^−/−^, and Vγ4^−/−^/Vγ6^−/−^ mice. **(A)** FACS analysis of IgA^+^ germinal center (GC) B cells (CD19^+^, CD138^−^, GL7^+^, CD95^+^, IgA^+^). **(B)** Quantification of IgA^+^ GC B cells (CD19^+^, CD138^−^, GL7^+^, CD95^+^, IgA^+^). Bar graph, mean ± SEM. n = 8–27 per group. Each dot represents an individual mouse. **(C, D)** Co-culture experiments of γδ T cells together with B cells isolated from PPs of *Tcrd*-H2BeGFP mice in the presence or absence of additional cytokines (100 ng/ml IL-4, 1 ng/ml IL-5, 1 ng/ml TGF-β) and 10 ng/ml LPS as indicated in the graphs. To all conditions 1 ng/ml IL-6 was added 3 days after the beginning of the culture. **(D)** FACS analysis was performed to quantify the percentage and absolute number of membrane IgA^+^ B cells (B220^+^, CD3^−^, IgM^−^, IgA^+^) cultured in presence or not of γδ T cells, LPS, and cytokines as indicated. Bar graph, mean ± SEM. n = 4–5. ANOVA test was applied with Tukey *post-hoc* test. *P < 0.05, **P < 0.01, ****P < 0.0001.

As most of the γδ T cells in PPs are located within the GCs, we next examined the influence of γδ T cell on GC B cells. To this end, we analyzed Vγ1^−/−^ mice, Vγ4^−/−^/Vγ6^−/−^ mice, non-depleted *Tcrd*-GDL mice, and *Tcrd*-GDL mice at 8 days, 3 weeks, and 8 weeks after γδ T cell depletion. We chose these three time-points, since 8 days after depletion no γδ T cells are present in the PPs, at 3 weeks after depletion the γδ T cells slowly start to regenerate, and 8 weeks after depletion, the percentage of γδ T cells is similar as before depletion, giving us the opportunity to compare their functions before and after regeneration (**Supplementary Figure 3A**). Interestingly, we found a reduction of IgA^+^ GC B cells in all mutant and depleted (8 days and 3 weeks) mice when compared to non-depleted *Tcrd*-GDL mice. Only after 8 weeks of depletion, when γδ T cells are regenerated, the IgA^+^ GC B cells increase again (**Figures 3A, B**), indicating that γδ T cells are important for the isotype switch towards IgA. However, the relative frequencies of GC B cells normalized to B cells in Vγ4^−/−^/Vγ6^−/−^ mice did not change when compared to non-depleted *Tcrd*-GDL control mice (**Supplementary Figure 5A**). Only in the Vγ1^−/−^ mice we found a significant reduction of this frequency (**Supplementary Figure 5A**), supporting the finding that Vγ1^+^/Vδ6.3/2^+^ T cells, as IL-4 producers, are important for isotype switch of GC B cells.

To further determine whether γδ T cells are important for the IgA class switch *in vitro*, we isolated B cells from the PPs and stimulated them with LPS, in the presence or absence of the IgA-inducing cytokines IL-4, IL-5, TGF-β, and IL-6 (**Figures 3C, D**) (48). In the presence of all cytokines, B cells are able to switch towards IgA, while in the absence of IL-4, this process was compromised (**Figures 3C, D**). Interestingly, when γδ T cells were added to the culture, in the absence of IL-4, the number and the frequency of mature IgA^+^ B cells were statistically increased compared to the samples without γδ T cells, consistent with our *in vivo* findings (**Figures 3C, D**).

**Figure 4:**
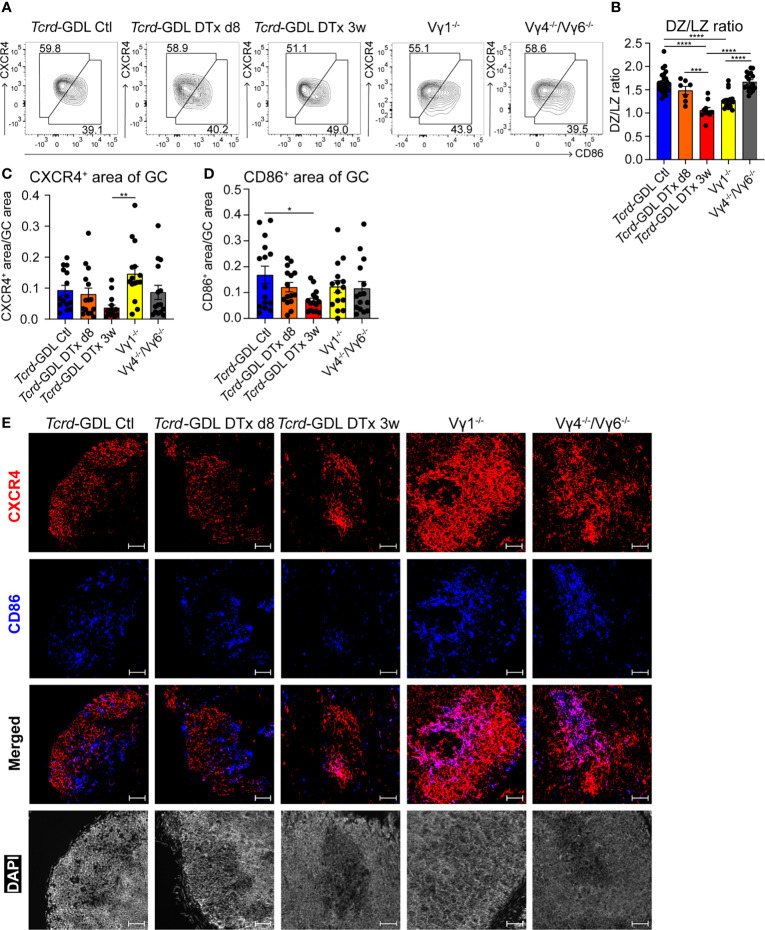
γδ T cells affect the structure of GCs. **(A)** FACS analysis of dark zone (DZ; CD19^+^, CD138^−^, GL7^+^, CD95^+^, CXCR4^high^, CD86^low^) and light zone (LZ; CD19^+^, CD138^−^, GL7^+^, CD95^+^, CXCR4^low^, CD86^high^) GC B cells of PPs. **(B)** Quantification of DZ/LZ ratio. Bar graph, mean ± SEM. n = 8–27 per group. ANOVA test was applied with Tukey *post-hoc* test. **(C, D)** Quantification of CXCR4 and CD86 area of GCs. For the quantification, sections from the proximal, medial, and distal part of the PPs were analyzed for each mouse. Each dot represents an individual cut. Bar graphs, mean ± SEM. n = 5 mice per group. ANOVA test was applied with Tukey *post-hoc* test. *P < 0.05, **P < 0.01, ***P < 0.001, ****P < 0.0001. **(E)** Fluorescence microscopy of frozen sections of PPs from non-depleted and γδ T cell–depleted (8 days and 3 weeks) *Tcrd*-GDL, Vγ1^−/−^, and Vγ4^−/−^/Vγ6^−/−^ mice stained with anti-CXCR4 (red), anti-CD86 (blue), and DAPI (white) to detect the nuclei. Scale bar, 50 µm. All sections were handled and treated similarly, and all pictures were acquired using the same settings.

Next, we examined the structure of the DZ and the LZ of the GCs by using two different markers, CXCR4 for the DZ and CD86 for the LZ (21, 53). Three weeks after γδ T cell depletion, *Tcrd*-GDL mice showed a decrease of the DZ/LZ ratio to a value of 1.0 compared to Vγ4^−/−^/Vγ6^−/−^ mice, non-depleted *Tcrd*-GDL mice, and 8-day depleted *Tcrd*-GDL mice, which all presented a DZ/LZ ratio of approximately 1.5. At the same time, Vγ1^−/−^ mice presented a milder reduction of DZ/LZ ratio compared to 3-week depleted *Tcrd*-GDL mice (**Figures 4A, B**).

To further corroborate the changes in the DZ and the LZ of the GCs observed after γδ T cell depletion, we performed immunofluorescence staining with antibodies directed against CXCR4 and CD86 in sections from the proximal, medial, and distal parts of the PPs. Automated analysis of the pictures confirmed a significant reduction of CD86^+^ GC B cells (LZ) in 3 weeks after γδ T cell depletion *Tcrd*-GDL mice (**Figures 4D, E**), confirming the data obtained by FACS analysis (**Figures 4A, B**). On the other hand, Vγ1^−/−^ mice presented an increase in the DZ area (**Figures 4C, E**). Moreover, while in untreated control *Tcrd*-GDL mice the border of the DZ and LZ were in most of the cases discernible, in Vγ1^−/−^ and Vγ4^−/−^/Vγ6^−/−^ mice, the two areas overlapped, thus altering the spatial organization of the GCs (**Figure 4E**). These data indicate a role for γδ T cells in keeping the structure of the GCs.

As the DZ and the LZ structure of GCs was altered in the absence of γδ T cells, we tested whether this could alter the size of the GCs. However, when we analyzed sections of PPs from depleted and non-depleted *Tcrd*-GDL, Vγ1^−/−^, and Vγ4^−/−^/Vγ6^−/−^ mice, the GC size was comparable among all groups (**Supplementary Figures 5B, C**).

FDCs have an essential role in keeping GC structure by providing high quantities of CXCL13, which is important for the correct positioning of LZ GC B cells (16, 17, 19). However, we did not find any differences in the amount and localization of FDCs by using FDC-M1 and CD35 as markers, among all groups (**Supplementary Figures 6**, **7**).

Finally, to exclude the possibility that DTx treatment alone would affect B cell dynamics in PPs, we examined GC B cells (**Supplementary Figure 3B**), IgA^+^ GC B cells (**Supplementary Figure 3C**), and DZ/LZ ratio (**Supplementary Figure 3D**) in DTx-injected *Tcrd*-H2BeGFP mice, which are unresponsive to DTx. However, we found no differences in control and injected *Tcrd*-H2BeGFP mice (8 days and 3 weeks after the injection).

Taken together, these data further support the hypothesis that γδ T cells are important for the isotype switch of GC B towards IgA *via* production of IL-4 and for keeping up the structure of the GCs.

### GC B Cells Proliferate Less in γδ T Cell–Depleted Mice

**Figure 5:**
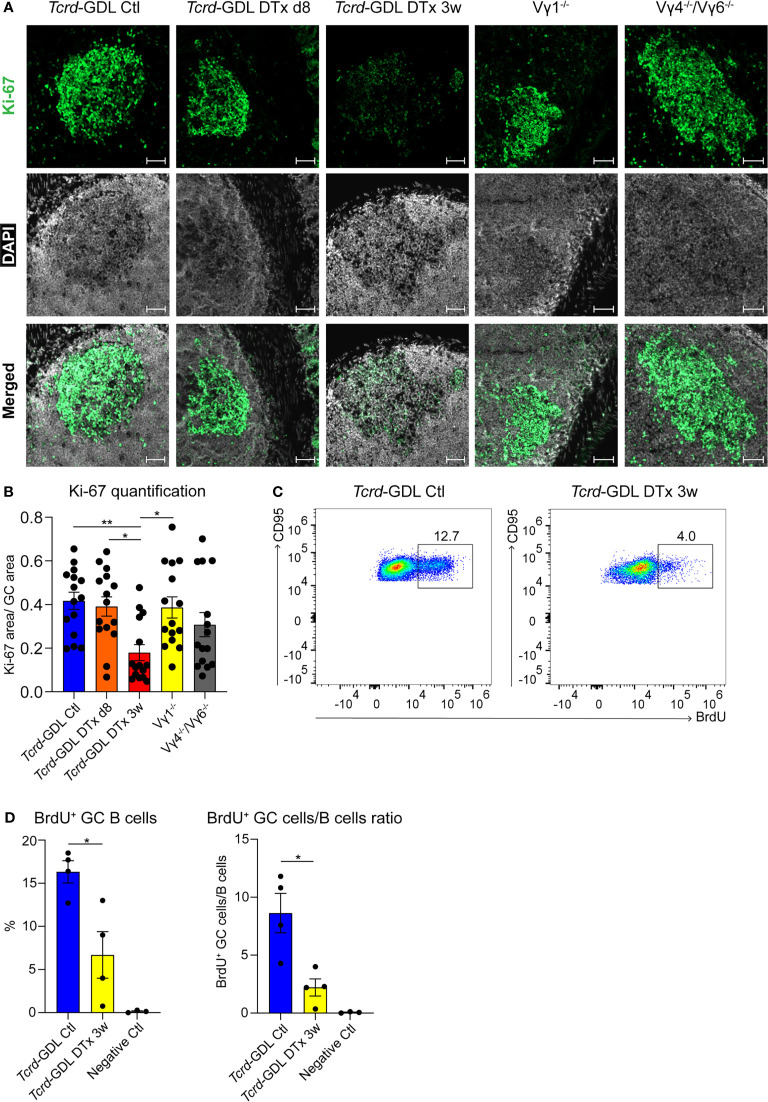
Decreased proliferation in the DZ of GCs after γδ T cell depletion. **(A)** Fluorescence microscopy of frozen sections of PPs from non-depleted and γδ T cell–depleted (8 days and 3 weeks) *Tcrd*-GDL, Vγ1^−/−^, and Vγ4^−/−^/Vγ6^−/−^ mice stained with anti-Ki-67 (green) and DAPI (white) to detect the nuclei. Scale bar, 50 µm. All sections were handled and treated similarly, and all pictures were acquired using the same settings. **(B)** For the quantification, sections from the proximal, medial, and distal part of the PPs were analyzed for each mouse. Each dot represents an individual cut. Bar graph, mean ± SEM. n = 5 mice per group. ANOVA test was applied with Tukey *post-hoc* test. **(C, D)** Non-depleted and 3-week γδ T cell–depleted *Tcrd*-GDL mice were injected with bromodeoxyuridine (BrdU) and analyzed 2 h after injection. **(C)** FACS analysis of BrdU^+^ GC B cells gated on GC B cells (CD19^+^, CD138^−^, GL7^+^, CD95^+^) from PPs. **(D)** Quantification of BrdU^+^ GC B cells (CD19^+^, CD138^−^, GL7^+^, CD95^+^, BrdU^+^). Bar graphs, mean ± SEM. n = 4 mice per group. T-test was applied. Each dot represents an individual mouse. *P < 0.05, **P < 0.01.

To investigate possible mechanisms underlying the reduction of IgA^+^ GC B cells and the change in the structure of the GCs, we analyzed the proliferation of cells within the GCs by the expression of Ki-67. Proliferation and cell division is classically restricted to the DZ of GCs (54, 55), but under certain conditions it can also appear in the LZ of GCs, especially in mice (20, 21, 56). Ki-67 expression was severely reduced 3 weeks after γδ T cell depletion in *Tcrd*-GDL mice, whereas in Vγ1^−/−^ and Vγ4^−/−^/Vγ6^−/−^ mice we did not find any significant differences compared to non-depleted *Tcrd*-GDL mice (**Figures 5A, B**). However, in Vγ1^−/−^ and Vγ4^−/−^/Vγ6^−/−^ mice, as previously highlighted, the separation between the DZ and the LZ was impaired (**Figure 4E**). To further determine whether GC B cell proliferation was altered by the absence of γδ T cells, we quantified GC B cells that incorporate the thymidine analog BrdU during 2 h pulse in 3-week γδ T cell–depleted and non-depleted *Tcrd*-GDL mice (**Figures 5C, D**). Significantly fewer BrdU^+^ GC B cells were present in the depleted mice when compared to the non-depleted mice, indicating that indeed γδ T cells have an important role in controlling GC B cell proliferation (**Figure 5C, D**).

We next sought to test whether a direct interaction between the γδ TCR and B cells could be responsible for the changes in the GCs observed above. Therefore, we injected *Tcrd*-H2BeGFP mice once a week for 5 weeks with antibodies directed against the γδ TCR (clone GL3). No differences could be detected between injected and control mice for IgA^+^ GC B cells (**Supplementary Figure 8A**) as well as in the DZ/LZ ratio (**Supplementary Figure 8B**). This implies that a direct interaction between γδ TCR and B cells is not responsible for the alteration of IgA^+^ GC B cells and the structural changes of the GCs.

### γδ T Cells Support the Formation of GC B Cells During *Salmonella* Infection

**Figure 6:**
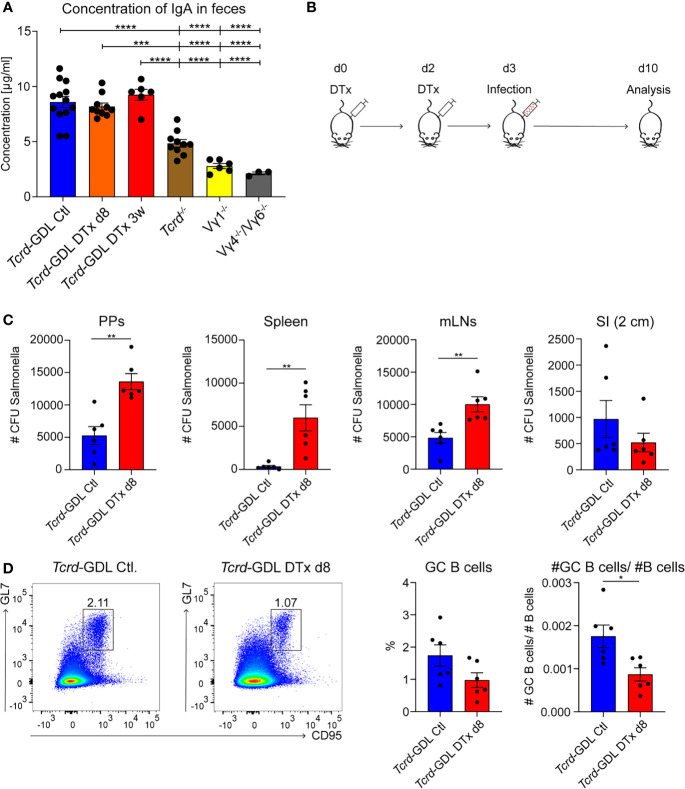
γδ T cells contribute to the immune response against *Salmonella* infection. **(A)** Concentration of IgA isolated from feces of non-depleted and γδ T cell–depleted (8 days and 3 weeks) *Tcrd*-GDL, *Tcrd* ^−/−^, Vγ1^−/−^, and Vγ4^−/−^/Vγ6^−/−^ mice were measured by ELISA. Bar graph, mean ± SEM. n = 3–12 per group. ANOVA test was applied with Tukey *post-hoc* test. **(B)** Non-depleted or depleted *Tcrd*-GDL mice were orally infected for 7 days with *Salmonella* and analyzed at day 10. **(C, D)** Non-depleted *Tcrd*-GDL or depleted *Tcrd*-GDL mice were orally infected for 7 days with *Salmonella*. **(C)** Quantification of colony-forming units (CFUs) from PPs, spleen, mesenteric lymph nodes (mLNs), and small intestine (SI) of non-depleted and γδ T cell–depleted (8 days) *Tcrd*-GDL mice. Bar graphs, mean ± SEM. n = 5–6 per group. T-test was applied. **(D)** Analysis of germinal center (GC) B cells (CD19^+^, CD138^−^, GL7^+^, CD95^+^) of non-depleted and γδ T cell–depleted (8 days) *Tcrd*-GDL mice after *Salmonella* infection. Bar graphs, mean ± SEM. n = 6 per group. T-test was applied. Each dot represents an individual mouse. *P < 0.05, **P < 0.01, ***P < 0.001, ****P < 0.0001.

As IgA^+^ GC B cells were reduced in absence of γδ T cells, we investigated the concentration of IgA in the feces of *Tcrd* ^−/−^, Vγ1^−/−^, Vγ4^−/−^/Vγ6^−/−^, depleted, and non-depleted *Tcrd*-GDL mice. IgA was significantly decreased in Vγ1^−/−^, Vγ4^−/−^/Vγ6^−/−^, and *Tcrd* ^−/−^ mice (**Figure 6A**), corroborating the finding that γδ T cells contribute to the production of IgA in PPs. To further determine the role of γδ T cells in the IgA-dependent immune response to an infection with a gut-associated pathogen, we used the *Salmonella* enteric mouse model. The SL1344ΔaroA strain of *S. typhimurium*, which has a metabolic mutation that attenuates its virulence, was selected for this purpose (57). One day after γδ T cell depletion or mock depletion, *Tcrd*-GDL mice were infected with *S. typhimurium* and analyzed 7 days after the infection (**Figure 6B**). γδ T cell–depleted *Tcrd-*GDL mice exhibited significantly higher bacterial burden compared to the non-depleted mice in all the analyzed organs (PPs, spleen, and mLNs) with the exception for SI (**Figure 6C**), indicating that γδ T cells can reduce the bacterial spread and clearance into different organs. Moreover, analysis of GC B cells 7 days after the infection showed a reduction in the frequency and absolute number of GC B cells in γδ T cell–depleted *Tcrd*-GDL mice when compared to the non-depleted mice (**Figure 6D**).

Together, these data indicate that γδ T cells are important for mounting a proper humoral immune response against *S. typhimurium*.

## Discussion

The GC reactions require a coordinated interplay between several cell types and cytokines to efficiently generate mature B cells producing high-affinity IgA (4, 22, 58). So far, several studies showed the influence of γδ T cells in spleens and LNs on the GC reactions, resulting in changed Ig isotypes, antibody production, and TFH cell development (28, 59–61). However, the importance of γδ T cells for mucosal immune responses in PPs was not yet systematically investigated.

Here we found that Vγ1^+^ T cells represent the biggest subset of γδ T cells in PPs. A great amount of these Vγ1^+^ T cells co-expressed the Vδ6.3/2 chain and produced IL-4, which promotes the isotype switch of GC B cells towards IgA. This specific subset was previously characterized as mainly resident in spleen and liver, where it expressed a very restricted TCR repertoire (37).

Several studies highlighted the central role of IL-4 in CSR to IgA (11–13) and proliferation of GCs (62, 63). So far, this important cytokine was thought to be mainly produced by TFH cells in PPs (19, 64, 65); however, we did not find any significant differences in this T cell subtype. Probably, both γδ T and TFH cells are the great contributors to the production of IL-4 in GCs. It has been described that in the LNs, γδ T cells could help TFH cells during their maturation through the release of Wnt ligands (61). It was thus conceivable to find a similar function in PPs; however, TFH cells in PPs develop from different progenitors than in other secondary lymphoid organs (66, 67). Moreover, GCs in PPs are constitutively formed due to microbiota stimulation (68, 69), underlying the profound differences between PPs and other secondary lymphoid organs. Still, TFH cells have crucial roles in the production of antigen-specific antibodies, GC reactions, and production of IL-4.

In the absence of αβ T cells, GC reactions are still occurring. Indeed, upon infection, GCs can be found in *Tcrb* ^−/−^ mice, which lack αβ T cells but not γδ T cells (70). Another study could show that SHM appears with the same frequency in *Tcrb* ^−/−^ mice compared to control mice, highlighting that γδ T cells can also support SHM in the absence of αβ T cells (71). As for IgA, in non-immunized mice, their level, together with IgM and IgG, was decreased in Vγ1^−/−^ mice (41). Also in another study, the concentration of IgA in serum, saliva, and fecal samples was reduced in *Tcrd* ^−/−^ mice (72). Additionally, after oral immunization with cholera toxin and tetanus toxin, the reduction of IgA was even stronger when compared to control mice. Interestingly, for IgM and IgG levels, no effects could be observed, indicating a specific role of γδ T cells in PPs for the production of IgA (72).

In humans, a specific γδ T cell subset of “innate” Vγ9^+^/Vδ2^+^ T cells expresses CXCR5, co-stimulatory molecules (ICOS and CD40L), and produces IL-4, IL-10, and CXCL13 to help B cells in development and production of IgA, IgG, and IgM (73). Co-culture experiments of γδ T cells and B cells showed that the helping effect observed from γδ T cells is as high as from TFH cells (74–76). Interestingly, in patients who suffer from IgA nephropathy, there is a positive correlation between the proportion of IgA^+^ B cells and γδ T cells. The enhancement of IgA was abolished after removal of γδ T cells (77). Therefore, it is not surprising that besides αβ T cells, γδ T cells play an important role in helping B cell maturation and switching to IgA in GC reactions.

In summary, our data lead to a better understanding of the role of γδ T cells in PPs GC reactions. We propose that Vγ1^+^/Vδ6^+^ T cells produce large amounts of IL-4 in PPs of mice and specifically support CSR to IgA and proliferation of GC B cells in mice. It remains to be determined whether Vγ1^+^/Vδ6^+^ T cells need to recognize specific signals *via* their TCR or whether this help is independent of cognate γδ TCR antigen. In sum, γδ T cells in PPs help B cells to establish and sustain the GC reactions and thereby, synergistically with local αβ T cells, maintain the symbiosis of the gut and its microbiota and contribute to IgA-dependent immune responses against intestinal pathogens.

## Data Availability Statement

The original contributions presented in the study are publicly available. This data can be found here: http://www.ncbi.nlm.nih.gov/bioproject/741071.

## Ethics Statement

The animal study was reviewed and approved by Lower Saxony State Office for Consumer Protections and Food Safety. Written informed consent was obtained from the owners for the participation of their animals in this study.

## Author Contributions

LU designed and performed the experiments, analyzed data, and wrote the manuscript. YL and GEP analyzed data. AW, A-LJ, JB-M, AB, AD, and AJ performed experiments. KI and IS provided essential reagents. IP supervised the project, including the experimental design, and edited the manuscript. FR supervised the project, designed and performed the experiments, analyzed data, and wrote the manuscript. All authors contributed to the article and approved the submitted version.

## Funding

The researchers received funding from the Medical School of Hannover (HILF I-Hochschulinterne Leistungsförderung), number 79228008 (to FR), and from the Deutsche Forschungsgemeinschaft, grants PR727/8-1 and PR727/13-1 (to IP).

## Conflict of Interest

The authors declare that the research was conducted in the absence of any commercial or financial relationships that could be construed as a potential conflict of interest.

## Publisher’s Note

All claims expressed in this article are solely those of the authors and do not necessarily represent those of their affiliated organizations, or those of the publisher, the editors and the reviewers. Any product that may be evaluated in this article, or claim that may be made by its manufacturer, is not guaranteed or endorsed by the publisher.
