# Supplementary material for: IL-4-Producing Vγ1+/Vδ6+ γδ T Cells Sustain Germinal Center Reactions in Peyer’s Patches of Mice
Source: Front Immunol. 2021 Nov 3;12:729607. doi: 10.3389/fimmu.2021.729607 (PMC8600568; doi:10.3389/fimmu.2021.729607)
Supplement: Supplementary file 1 [file DataSheet_1.pdf]

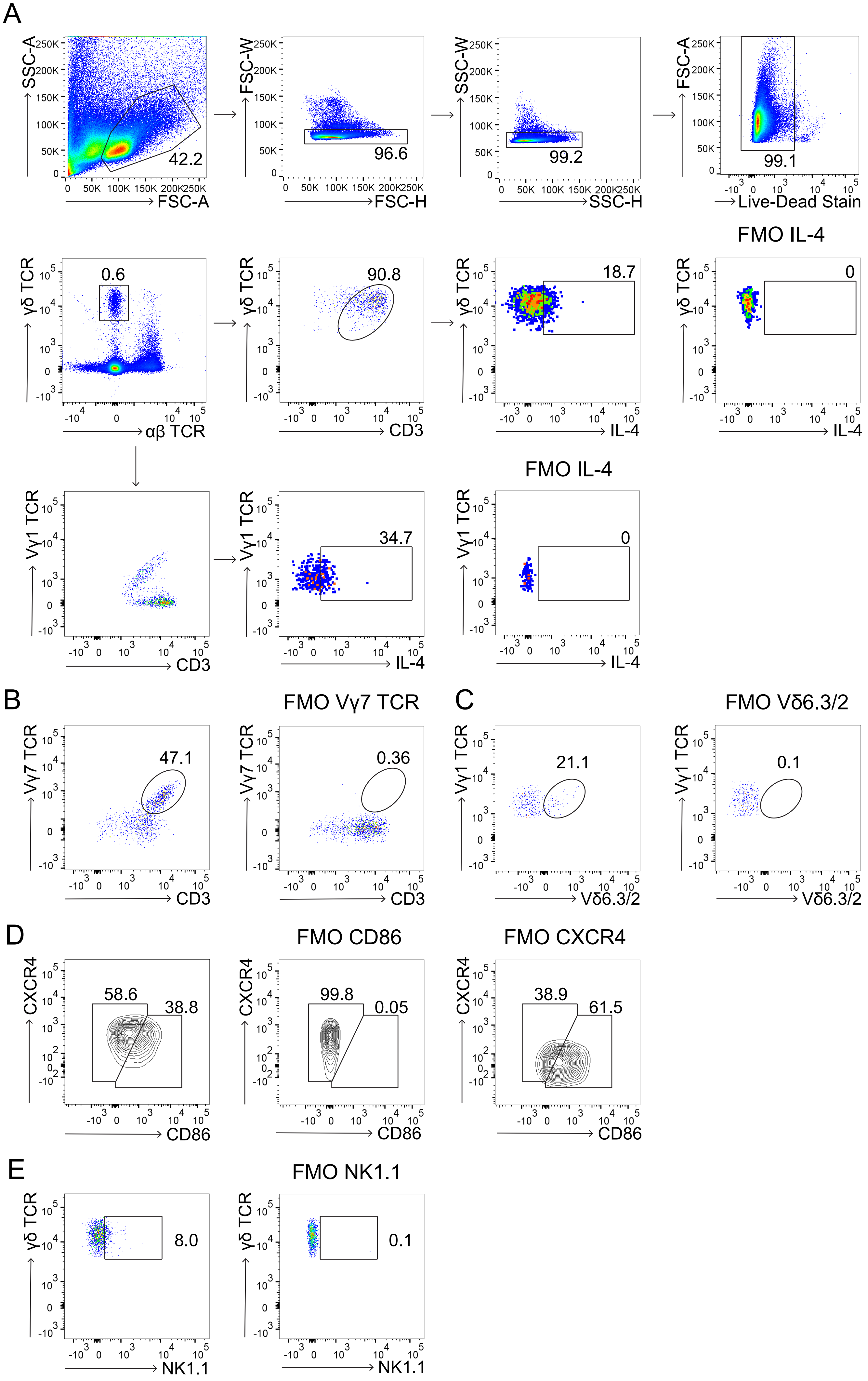
A
SSC-A
FSC-A
42.2
FSC-W
FSC-H
96.6
SSC-W
SSC-H
99.2
FSC-A
Live-Dead Stain
99.1
γδ TCR
αβ TCR
0.6
γδ TCR
CD3
90.8
γδ TCR
IL-4
18.7
FMO IL-4
γδ TCR
IL-4
0
Vγ1 TCR
CD3
Vγ1 TCR
IL-4
34.7
FMO IL-4
Vγ1 TCR
IL-4
0
B
Vγ7 TCR
CD3
47.1
FMO Vγ7 TCR
Vγ7 TCR
CD3
0.36
C
Vγ1 TCR
Vδ6.3/2
21.1
FMO Vδ6.3/2
Vγ1 TCR
Vδ6.3/2
0.1
D
CXCR4
CD86
58.6
38.8
FMO CD86
CXCR4
CD86
99.8
0.05
FMO CXCR4
CXCR4
CD86
38.9
61.5
E
γδ TCR
NK1.1
8.0
FMO NK1.1
γδ TCR
NK1.1
0.1

A

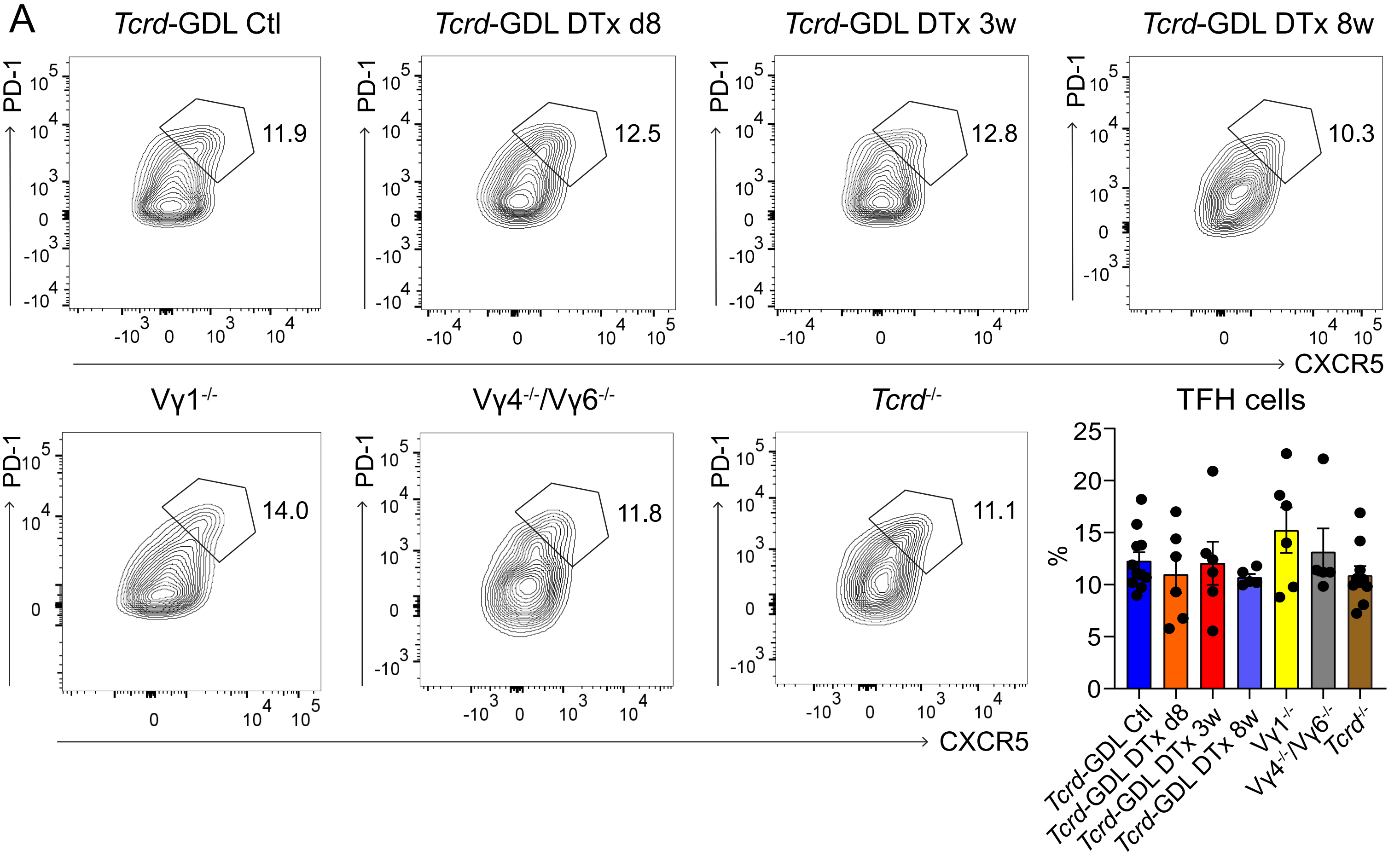
Tcrd-GDL Ctl
Tcrd-GDL DTx d8
Tcrd-GDL DTx 3w
Tcrd-GDL DTx 8w
11.9
12.5
12.8
10.3
Vγ1-/-
Vγ4-/-/Vγ6-/-
Tcrd-/-
14.0
11.8
11.1
PD-1
CXCR5
TFH cells
%

B

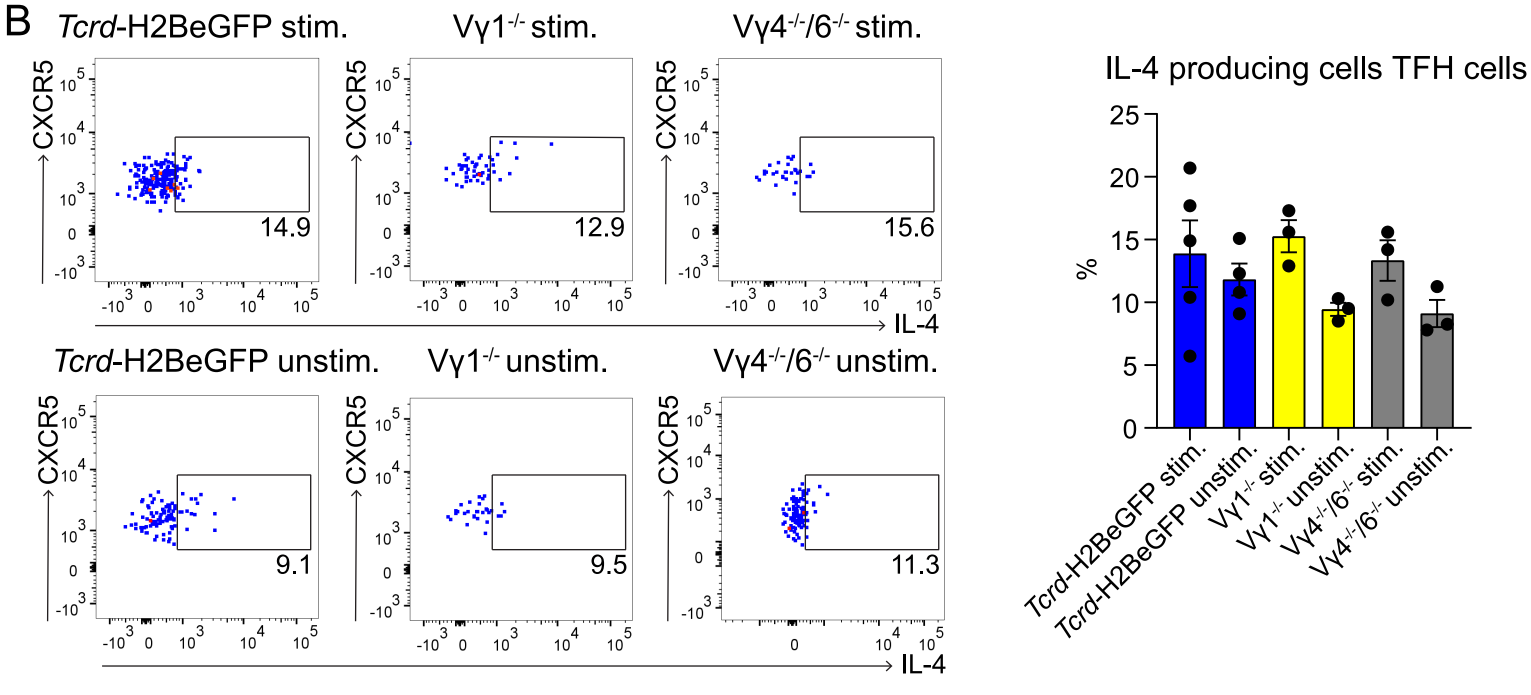
Tcrd-H2BeGFP stim.
Vγ1-/- stim.
Vγ4-/-/6-/- stim.
14.9
12.9
15.6
Tcrd-H2BeGFP unstim.
Vγ1-/- unstim.
Vγ4-/-/6-/- unstim.
9.1
9.5
11.3
CXCR5
IL-4
IL-4 producing cells TFH cells
%

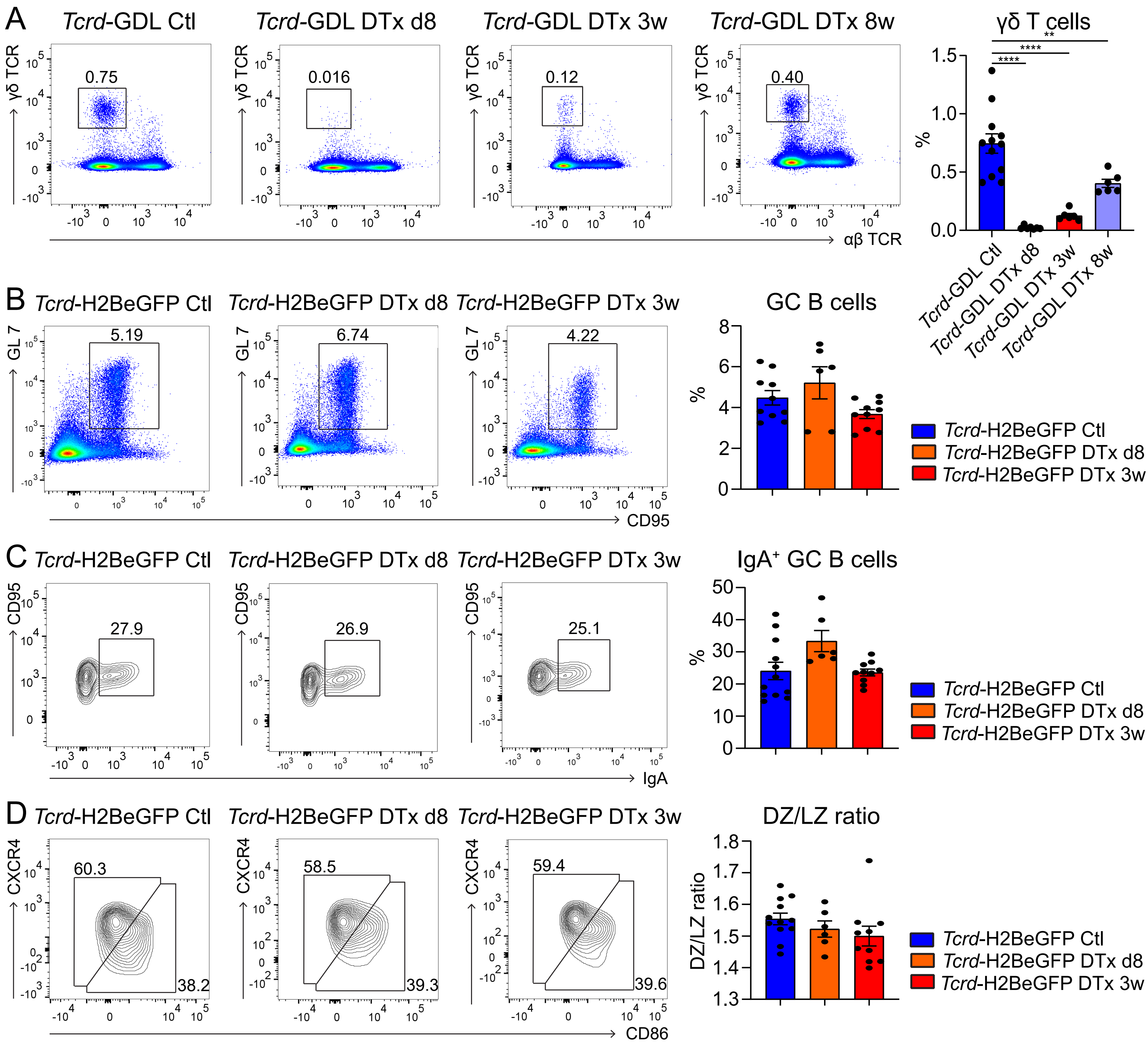

A
Tcrd-GDL Ctl
Tcrd-GDL DTx d8
Tcrd-GDL DTx 3w
Tcrd-GDL DTx 8w
γδ TCR
αβ TCR
0.75
0.016
0.12
0.40
γδ T cells
%
**
****
****
B
Tcrd-H2BeGFP Ctl
Tcrd-H2BeGFP DTx d8
Tcrd-H2BeGFP DTx 3w
GL 7
CD95
5.19
6.74
4.22
GC B cells
C
CD95
IgA
27.9
26.9
25.1
IgA+ GC B cells
D
CXCR4
CD86
60.3
38.2
58.5
39.3
59.4
39.6
DZ/LZ ratio

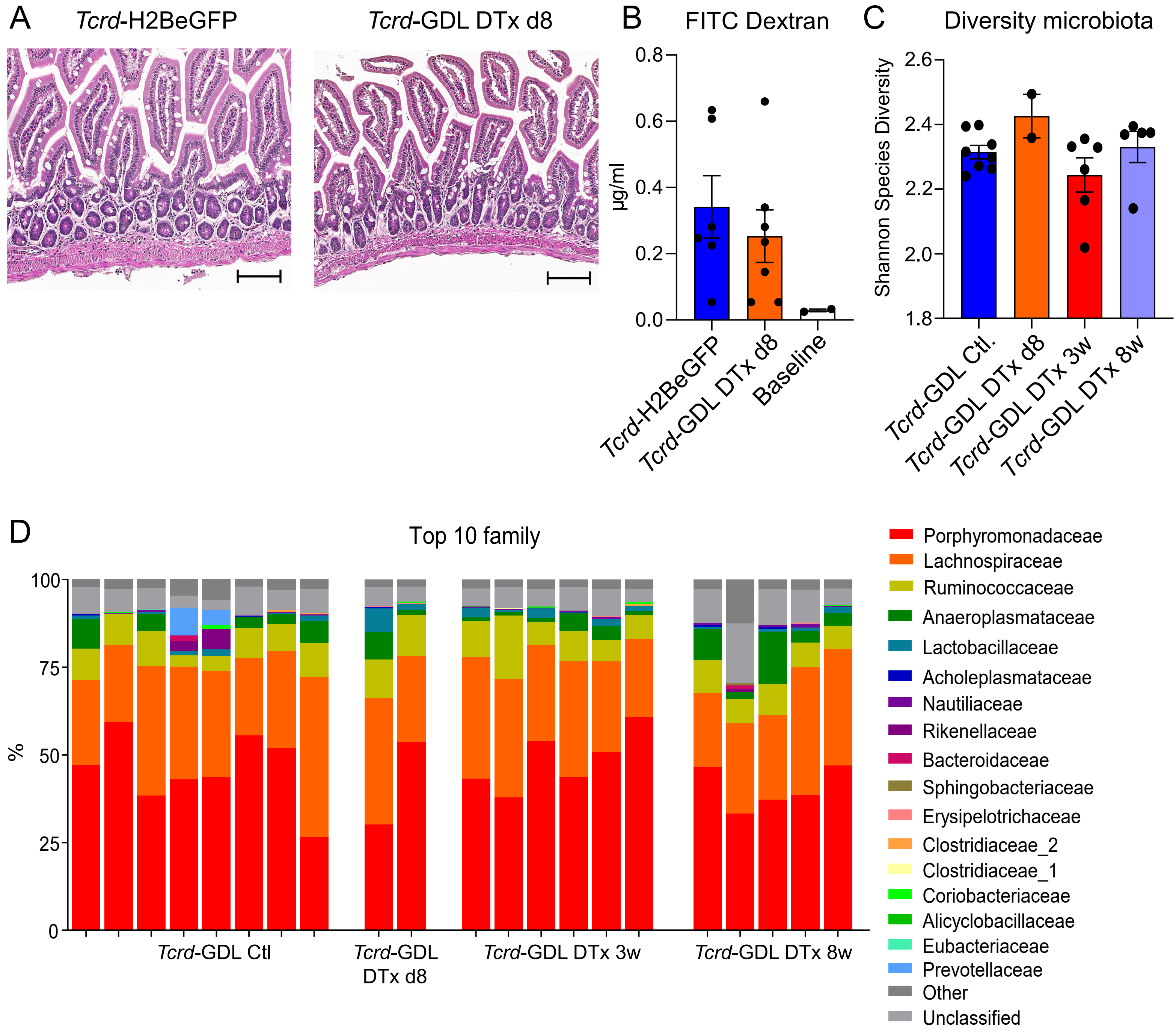
A
Tcrd-H2BeGFP
Tcrd-GDL DTx d8
B
FITC Dextran
μg/ml
0.8
0.6
0.4
0.2
0.0
Tcrd-H2BeGFP
Tcrd-GDL DTx d8
Baseline
C
Diversity microbiota
Shannon Species Diversity
2.6
2.4
2.2
2.0
1.8
Tcrd-GDL Ctl.
Tcrd-GDL DTx d8
Tcrd-GDL DTx 3w
Tcrd-GDL DTx 8w
D
Top 10 family
%
100
75
50
25
0
Tcrd-GDL Ctl
Tcrd-GDL DTx d8
Tcrd-GDL DTx 3w
Tcrd-GDL DTx 8w
Porphyromonadaceae
Lachnospiraceae
Ruminococcaceae
Anaeroplasmataceae
Lactobacillaceae
Acholeplasmataceae
Nautiliaceae
Rikenellaceae
Bacteroidaceae
Sphingobacteriaceae
Erysipelotrichaceae
Clostridiaceae_2
Clostridiaceae_1
Coriobacteriaceae
Alicyclobacillaceae
Eubacteriaceae
Prevotellaceae
Other
Unclassified

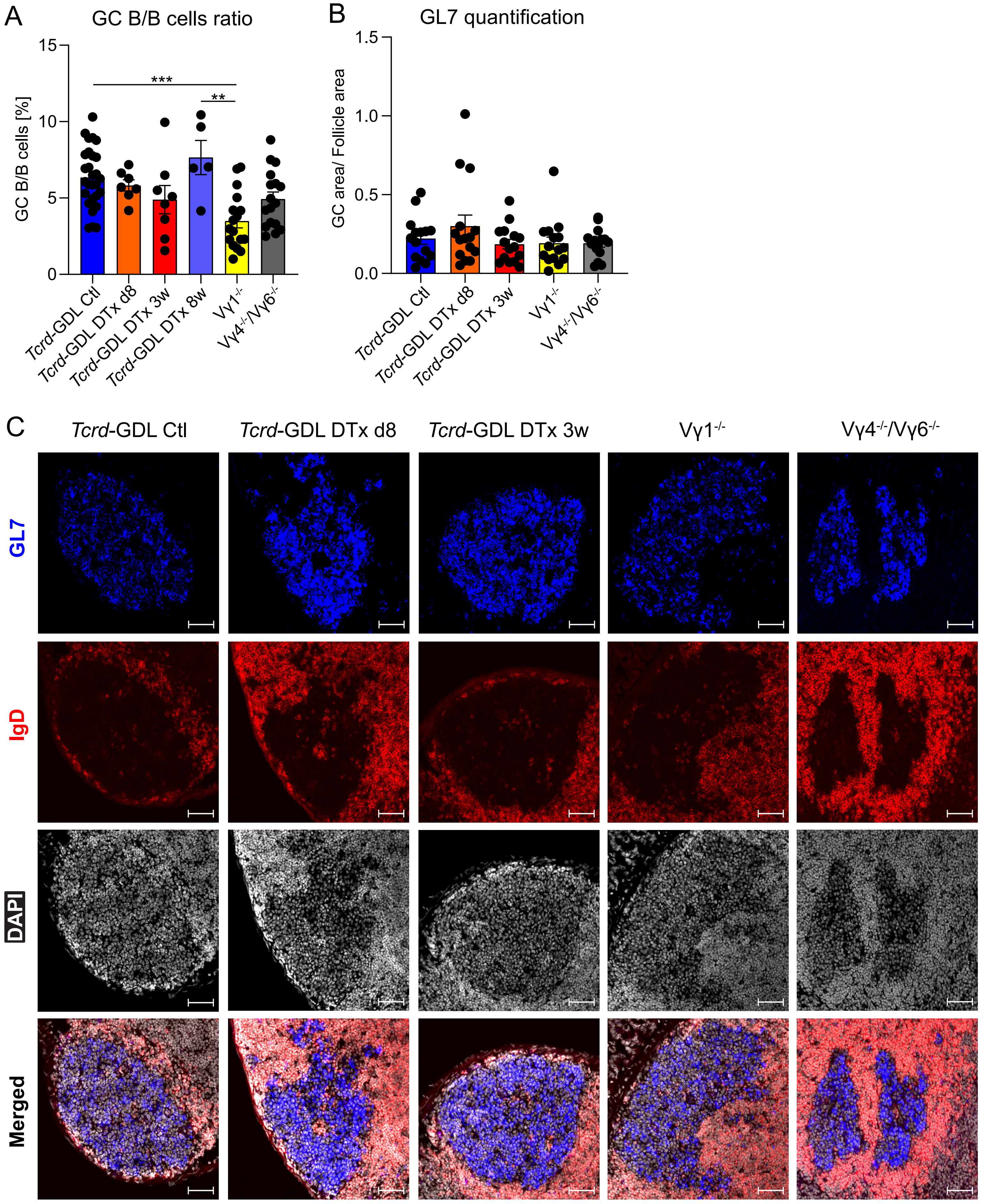
A
GC B/B cells ratio
GC B/B cells [%]
15
10
5
0
***
**
Tcrd-GDL Ctl
Tcrd-GDL DTx d8
Tcrd-GDL DTx 3w
Tcrd-GDL DTx 8w
Vγ1-/-
Vγ4-/-/Vγ6-/-
B
GL7 quantification
GC area/ Follicle area
1.5
1.0
0.5
0.0
Tcrd-GDL Ctl
Tcrd-GDL DTx d8
Tcrd-GDL DTx 3w
Vγ1-/-
Vγ4-/-/Vγ6-/-
C
Tcrd-GDL Ctl
Tcrd-GDL DTx d8
Tcrd-GDL DTx 3w
Vγ1-/-
Vγ4-/-/Vγ6-/-
GL7
IgD
DAPI
Merged

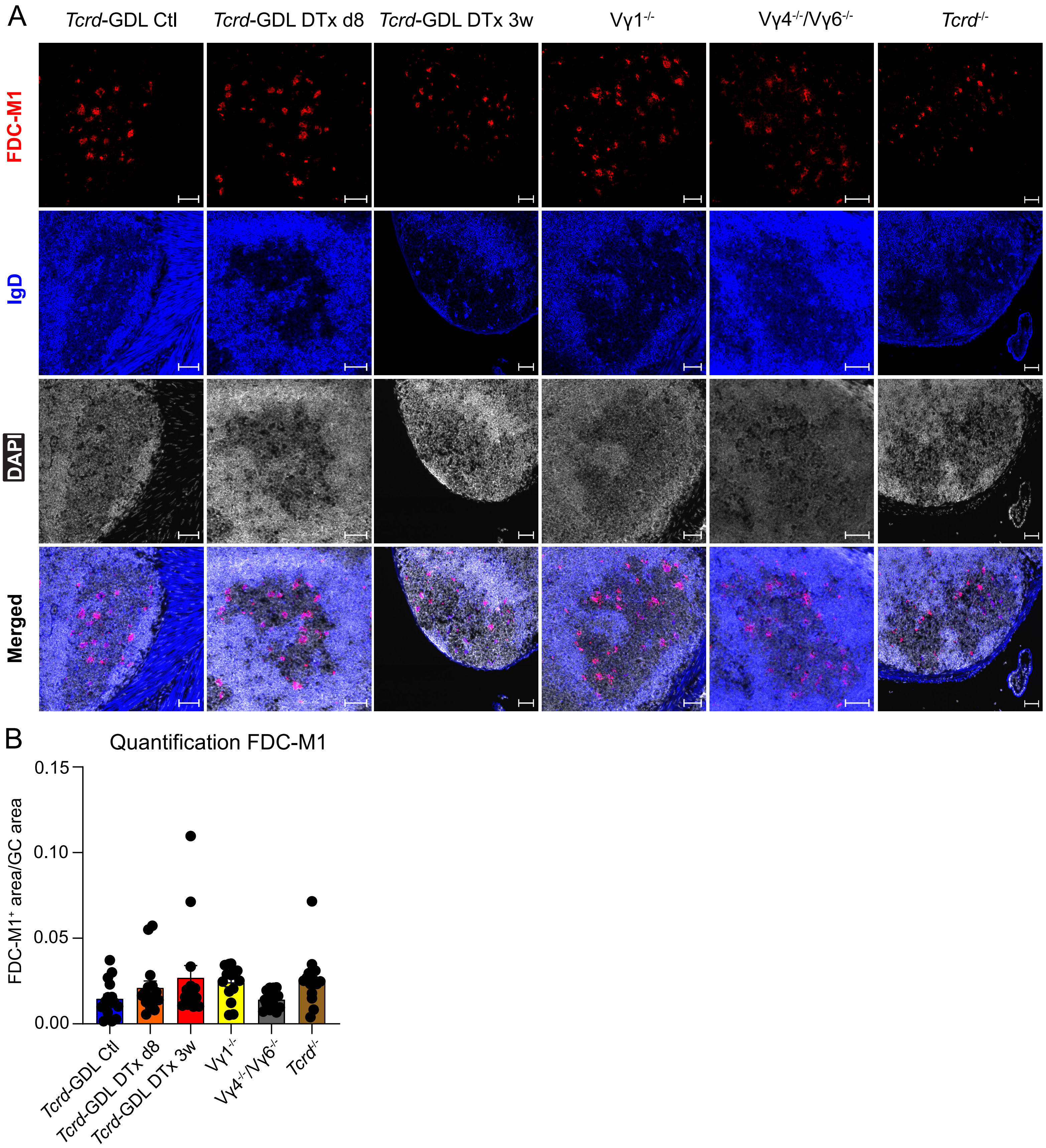
A
Tcrd-GDL Ctl
Tcrd-GDL DTx d8
Tcrd-GDL DTx 3w
Vγ1-/-
Vγ4-/-/Vγ6-/-
Tcrd-/-
FDC-M1
IgD
DAPI
Merged
B
Quantification FDC-M1
FDC-M1+ area/GC area
0.15
0.10
0.05
0.00
Tcrd-GDL Ctl
Tcrd-GDL DTx d8
Tcrd-GDL DTx 3w
Vγ1-/-
Vγ4-/-/Vγ6-/-
Tcrd-/-

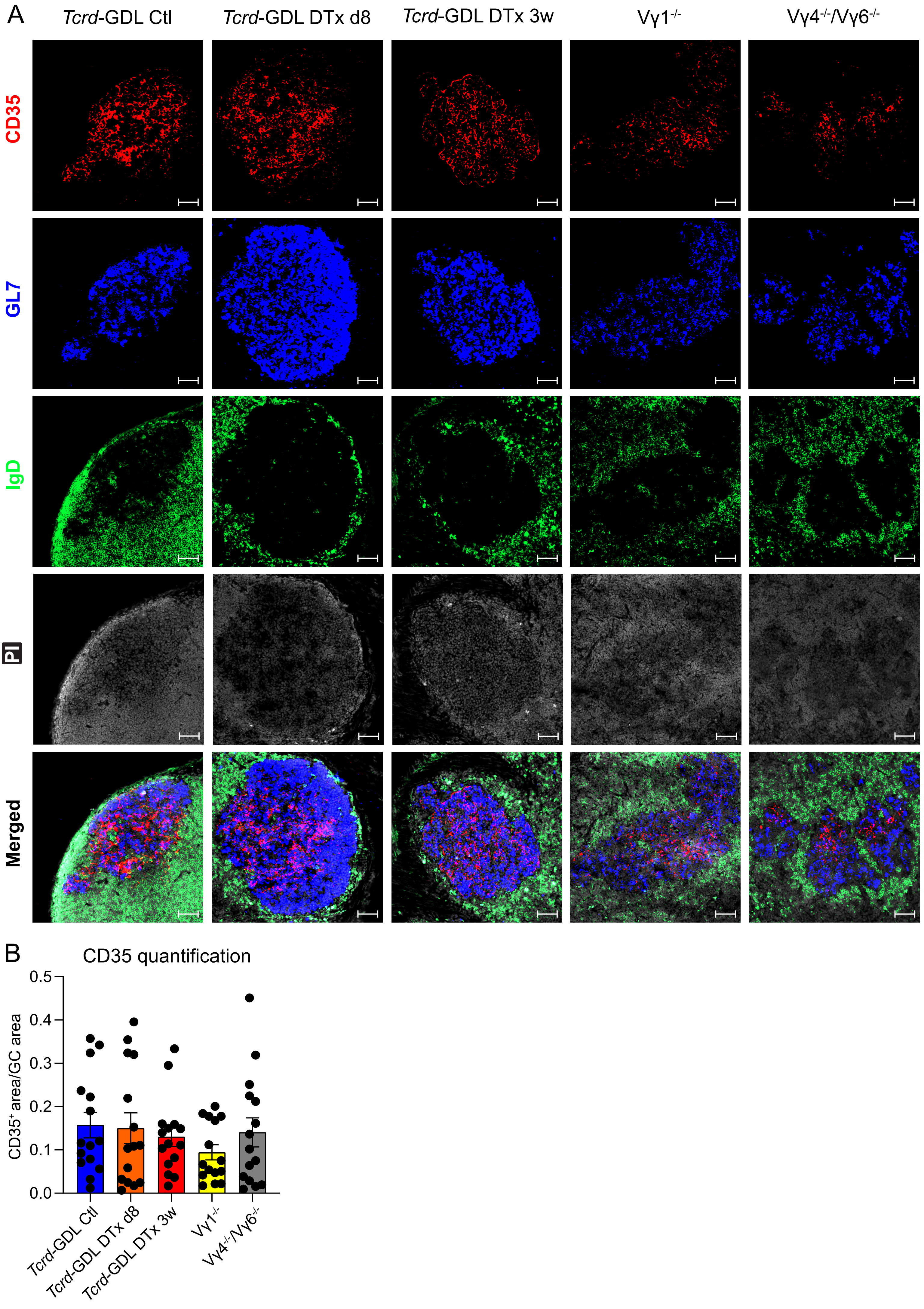
A
Tcrd-GDL Ctl
Tcrd-GDL DTx d8
Tcrd-GDL DTx 3w
Vγ1-/-
Vγ4-/-/Vγ6-/-
CD35
GL7
IgD
PI
Merged
B
CD35 quantification
CD35+ area/GC area
0.5
0.4
0.3
0.2
0.1
0.0
Tcrd-GDL Ctl
Tcrd-GDL DTx d8
Tcrd-GDL DTx 3w
Vγ1-/-
Vγ4-/-/Vγ6-/-

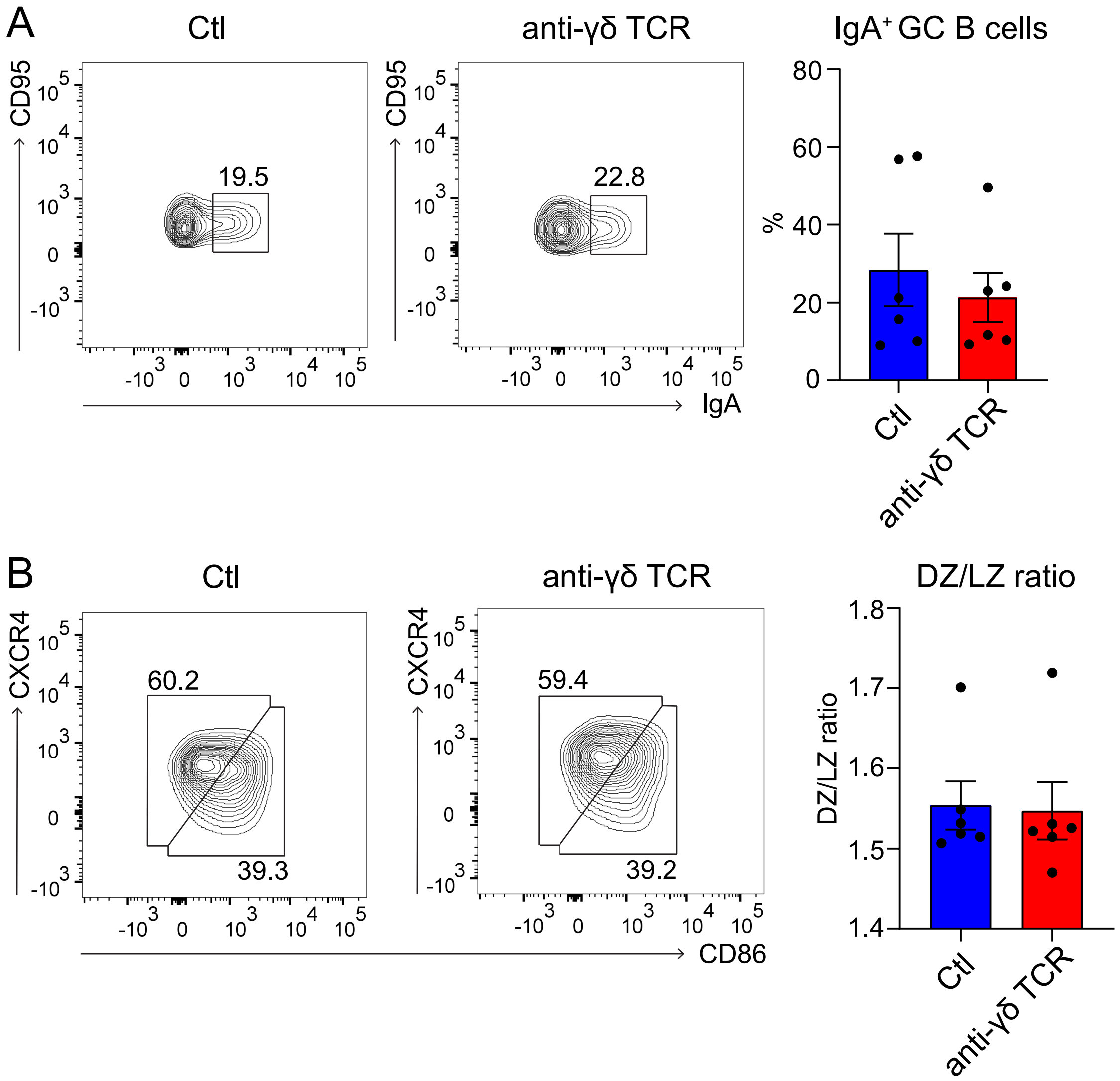
A
Ctl
anti-γδ TCR
IgA⁺ GC B cells
CD95
IgA
19.5
22.8
%
Ctl
anti-γδ TCR
B
Ctl
anti-γδ TCR
DZ/LZ ratio
CXCR4
CD86
60.2
39.3
59.4
39.2
DZ/LZ ratio
Ctl
anti-γδ TCR

Supplementary Material:

Supplementary Figure 1: Representative gating strategy for IL-4 assays and FMO controls.

(A) Representative gating strategy for interleukin-4 (IL-4) assay and fluorescence minus one (FMO) control for IL-4. **(B-D)** FMO controls of Vγ7 TCR gated on γδ T cells (TCRβ$^-$, CD3$^+$, GFP$^+$), Vδ6.3/2 gated on Vγ1$^+$ T cells (TCRβ$^-$, CD3$^+$, GFP$^+$, Vγ1 TCR$^+$), CD86 gated on germinal center (GC) B cells (CD19$^+$, CD138$^-$, GL7$^+$, CD95$^+$), CXCR4 gated on GC B cells (CD19$^+$, CD138$^-$, GL7$^+$, CD95$^+$), and NK1.1 gated on γδ T cells (TCRβ$^-$, CD3$^+$, GFP$^+$).

Supplementary Figure 2: TFH were not impaired after depletion of γδ T cells.

(A) FACS analysis of T follicular helper cells (TFH; CD4$^+$, TCRβ$^+$, CD3$^+$, CD44$^+$, PD-1$^+$, CXCR5$^+$) in PPs from non-depleted and γδ T cell depleted (eight days, three weeks, and eight weeks) *Tcrd*-GDL, Vγ1$^{-/-}$, Vγ4$^{-/-}$/Vγ6$^{-/}$, and *Tcrd*$^{-/-}$ mice. Bar graph, mean ± SEM. n = 5 - 10 per group. ANOVA test was applied with Tukey post-hoc test. (**B**) FACS analysis of interleukin-4$^+$ (IL-4$^+$) TFH cells in PPs of *Tcrd*-H2BeGFP, Vγ1$^{-/-}$, and Vγ4$^{-/-}$/ Vγ6$^{-/-}$ mice gated on TFH cells (CD4$^+$, TCRβ$^+$, CD3$^+$, CD44$^+$, PD-1$^+$, CXCR5$^+$). Cells were stimulated with mAbs directed against CD3 (1 µg/ml) and CD28 (1 µg/ml) for 2 hs and compared to the unstimulated controls. Bar graph, mean ± SEM. n = 3 – 4 per group. ANOVA test was applied with Tukey post-hoc test. Each dot represents an individual mouse.

Supplementary Figure 3: Injection of DTx specifically affects *Tcrd*-GDL mice and not control *Tcrd*-H2BeGFP mice.

(A) Analysis of γδ T cells (CD19$^-$, CD3$^+$, TCRβ$^-$, GFP$^+$) of PPs from non-depleted and γδ T cell depleted (eight days, three weeks, and eight weeks) *Tcrd*-GDL mice. Bar graphs, mean ± SEM. n = 6 - 12 per group. ANOVA test was applied with Tukey post-hoc test. **(B-D)** FACS analysis of non-injected and DTx injected (eight days and three weeks) *Tcrd*-H2BeGFP mice. **(B)** FACS analysis of germinal center (GC) B cells (CD19$^+$, CD138$^-$, GL7$^+$, CD95$^+$). Bar graph, mean ± SEM. n = 6 - 10 per group. ANOVA test was applied with Tukey post-hoc test. **(C)** FACS analysis of IgA$^+$ GC B cells (CD19$^+$, CD138$^-$, GL7$^+$, CD95$^+$, IgA$^+$) from PPs. Bar graph, mean ± SEM. n = 6 - 10 per group. ANOVA test was applied with Tukey post-hoc test. **(D)** FACS analysis of dark zone (DZ; CD19$^+$, CD138$^-$, GL7$^+$, CD95$^+$, CXCR4^{high}, CD86^{low}) and light zone (LZ; CD19$^+$, CD138$^-$, GL7$^+$, CD95$^+$, CXCR4^{low}, CD86^{high}) GC B cells from

PPs. Bar graph, mean ± SEM. n = 6 - 10 per group. ANOVA test was applied with Tukey post-hoc test. Each dot represents an individual mouse. **P < 0.01, ****P < 0.0001.

Supplementary Figure 4: Depletion of γδ T cells for eight days does not influence gut permeability and microbiota

(A) H/E staining of small intestine sections from *Tcrd*-H2BeGFP and eight day γδ T cell depleted *Tcrd*-GDL mice. Scale bar, 100 µm. **(B)** Quantification of fluorescein isothiocyanate dextran (FITC-d) in serum from *Tcrd*-H2BeGFP and γδ T cell depleted (eight days) *Tcrd*-GDL mice. Presence of FITC-d was assessed by ELISA. Baseline was calculated from sera of untreated mice. Bar graph, mean ± SEM. n = 3 - 6 per group. ANOVA test was applied with Tukey post-hoc test. **(C, D)** 16S metagenomic analysis of bacteria in feces from non-depleted and γδ T cell depleted (eight days, three weeks, and eight weeks) *Tcrd*-GDL mice. **(C)** Quantification of microbiota diversity by the Shannon index. Bar graph, mean ± SEM. n = 2 - 6 per group. ANOVA test was applied with Tukey post-hoc test. **(D)** Stacked bar charts showing the relative abundance of top 10 bacterial families from non-depleted and γδ T cell depleted (eight days, three weeks, and eight weeks) *Tcrd*-GDL mice.

Supplementary Figure 5: The size of the GCs was not influenced by depletion of γδ T cells.

(A) FACS analysis of germinal center (GC) B cells ($CD19^+$, $CD138^-$, $GL7^+$, $CD95^+$) compared to B cells ($CD19^+$, $CD138^-$) of PPs from non-depleted and γδ T cell depleted (eight days, three weeks, and eight weeks) *Tcrd*-GDL, $V\gamma1^{-/-}$, and $V\gamma4^{-/-}/V\gamma6^{-/-}$ mice. Bar graph, mean ± SEM. n = 8 - 27 per group. ANOVA test was applied with Tukey post-hoc test. Each dot represents an individual mouse. **(B, C)** Quantification of GL7 expression. For the quantification, sections from the proximal, medial, and distal part of the PPs were analysed for each mouse. Each dot represents an individual cut. Bar graph, mean ± SEM. n = 5 mice per group. ANOVA test was applied with Tukey post-hoc test. **(C)** Fluorescence microscopy of frozen sections of PPs from non-depleted and γδ T cell depleted (eight days and three weeks) *Tcrd*-GDL, $V\gamma1^{-/-}$, and $V\gamma4^{-/-}/V\gamma6^{-/-}$mice stained with anti-GL7 (blue), anti-IgD (red), and DAPI (white) to detect nuclei. Scale bar, 50 µm. All sections were handled and treated similarly and all pictures were acquired using the same settings. **P < 0.01, ****P < 0.0001.

Supplementary Figure 6: FDCs quantified by the marker FDC-M1 were not influenced by depletion of γδ T cells.

(A, B) Fluorescence microscopy of frozen sections of PPs from non-depleted and γδ T cell depleted (eight days and three weeks) *Tcrd*-GDL, $V\gamma1^{-/-}$, $V\gamma4^{-/-}/V\gamma6^{-/-}$, and *Tcrd*$^{-/-}$ mice stained with anti-FDC-M1 (red), anti-IgD (blue), and DAPI (white) to detect nuclei. Scale bar, 50 µm. All sections were handled and treated similarly and all pictures were acquired using the same settings. **(B)** Quantification of FDC-M1 expression. For the quantification, sections from the proximal, medial, and distal part of the PPs were analysed for each mouse. Each dot represents an individual cut. Bar graph, mean ± SEM. n = 5 mice per group. ANOVA test was applied with Tukey post-hoc test.

Supplementary Figure 7: FDCs quantified by the marker CD35 were not influenced by depletion of γδ T cells.

(A, B) Fluorescence microscopy of frozen sections of PPs from non-depleted and γδ T cell depleted (eight days and three weeks) *Tcrd*-GDL, $V\gamma1^{-/-}$, and $V\gamma4^{-/-}/V\gamma6^{-/-}$mice stained with anti-CD35 (red), anti-GL7 (blue), anti-IgD (green), and PI (white) to detect nuclei. Scale bar, 50 µm. All sections were handled and treated similarly and all pictures were acquired using the same settings. **(B)** Quantification of CD35 expression. For the quantification, sections from the proximal, medial, and distal part of the PPs were analysed for each mouse. Each dot represents an individual cut. Bar graph, mean ± SEM. n = 5 mice per group. ANOVA test was applied with Tukey post-hoc test.

Supplementary Figure 8: γδ TCR is not responsible for the observed changes in the GC

(A, B) *Tcrd*-H2BeGFP mice were injected with anti-γδ TCR antibody i.p. once a week for five weeks. PPs were isolated and FACS analysis was performed on control and anti-γδ TCR injected mice. **(A)** Analysis of IgA^+ germinal center (GC) B cells ($CD19^+$, $CD138^-$, $GL7^+$, $CD95^+$, IgA^+). Bar graph, mean ± SEM. n = 6 per group. ANOVA test was applied with Tukey post-hoc test. **(B)** Analysis of dark zone (DZ; $CD19^+$, $CD138^-$, $GL7^+$, $CD95^+$, $CXCR4^{high}$, $CD86^{low}$) and light zone (LZ; $CD19^+$, $CD138^-$, $GL7^+$, $CD95^+$, $CXCR4^{low}$, $CD86^{high}$) of PPs. Bar graph, mean ± SEM. n = 6 per group. ANOVA test was applied with Tukey post-hoc test. Each dot represents an individual mouse.

Supplementary Movie 1: Most of the γδ T cells localize in GCs of PPs

Confocal fluorescence microscopy of frozen sections of PPs from *Tcrd*-H2BeGFP mice stained with anti-IgD (red), anti-GL7 (orange) and DAPI (blue) to detect the nuclei. GFP-expressing γδ T cells are in green. Scale bar shown is 30 μm. Movie was created with 24 frames per second.
